# Parent- and child-reported executive functioning and response to psychotherapy in pediatric obsessive-compulsive disorder: Results from the TECTO study

**DOI:** 10.1007/s00787-026-03013-7

**Published:** 2026-03-28

**Authors:** Melanie Ritter, Valdemar Uhre, Sofie Heidenheim Christensen, Nicoline Løcke Jepsen Korsbjerg, Nicole Nadine Lønfeldt, Linea Pretzmann, Christine Lykke Thoustrup, Anna-Rosa Cecilie Mora-Jensen, Kerstin JessicaPlessen, Jens Richard Møllegaard Jepsen, Signe Vangkilde, Camilla Funch Uhre, Anne Katrine Pagsberg, Robert James Blair

**Affiliations:** 1 Child and Adolescent Mental Health Center, Copenhagen University Hospital – Bispebjerg and Frederiksberg, Gentofte Hospitalsvej 3A, Hellerup, Copenhagen 2900 Denmark; 2 Department of Clinical Medicine, Faculty of Health and Medical Sciences, University of Copenhagen, Blegdamsvej 3B, 33.5, Sektion A, København N, Copenhagen 2200 Denmark; 3 Danish Research Centre for Magnetic Resonance, Centre for Functional and Diagnostic Imaging and Research, Copenhagen University Hospital - Amager and Hvidovre, Kettegard Allé 30, Hvidovre, Copenhagen 2650 Denmark; 4 Department of Psychology, University of Copenhagen, Øster Farimagsvej 2A, København K, Copenhagen 1353 Denmark; 5 Mental Health Center Sct Hans, Capital Region of Denmark, Roskilde, Denmark; 6 Division of Child and Adolescent Psychiatry, Department of Psychiatry, Lausanne University Hospital (CHUV) and University of Lausanne, Av. d’Echallens 9, Lausanne, 1004 Switzerland; 7 Center for Clinical Neuropsychology, Children and Adolescents, Rigshospitalet, Blegdamsvej 9, København Ø, Copenhagen 2100 Denmark

**Keywords:** OCD, Executive Function, Children, Psychotherapy, Cognitive Behavioral Therapy

## Abstract

Executive function (EF) difficulties in pediatric obsessive-compulsive disorder (OCD) are suggested to interact with psychotherapy response. We investigated the role of parent- and self-rated EF in a randomized trial comparing cognitive-behavioral therapy (CBT) and psychoeducation with relaxation training (PRT). We investigated the extent to which: (1) EF difficulties are evident in non-medicated pediatric OCD patients; (2) difficulties improve during psychotherapy; and (3) EF moderates symptom alleviation. We included 114 patients with OCD (aged 8–17 years), who received 14 sessions of CBT or PRT. EF was assessed before and after treatment with both the parent-rated (full age span) and self-rated (age 11–17 years) Behavior Rating Inventory of Executive Function, 2nd version (BRIEF-2). All BRIEF-2 subdomains were reported. OCD symptom severity was assessed with the Children’s Yale-Brown Obsessive-Compulsive Scale (CY-BOCS). We assessed seventy-four non-psychiatric control children within a similar time interval. Parent- and self-ratings indicated EF difficulties in OCD patients compared with non-psychiatric controls across BRIEF-2 subdomains. Self-rated flexibility difficulties were associated with more severe OCD symptoms pre-treatment. Parents and children reported EF improvement after psychotherapy across subdomains. Parent-rated self-monitoring improved after CBT but not PRT. EF improvement was not associated with symptom alleviation in patients. Finally, emotional control, working memory, and task-completion ability moderated OCD-symptom improvement. EF difficulties are evident at the group level in pediatric OCD and show improvement following psychotherapy with minimal differences between CBT and PRT. Although EF improvement is not associated with symptom alleviation, several subdomains of EF pre-treatment moderate the treatment effect.

**Supplementary Information:**

The online version contains supplementary material available at 10.1007/s00787-026-03013-7.

## Introduction

### Obsessive-compulsive disorder in youth

Obsessive-compulsive disorder (OCD) is a debilitating psychiatric disorder that affects up to 3% of children and adolescents worldwide [2, 17]. OCD is characterized by intrusive, distressing, recurring thoughts/images (obsessions) and repetitive behavioral or mental rituals (compulsions) [7]. Psychiatric comorbidity is common in pediatric OCD (according to a recent meta-analysis 63.6%), with attention-deficit/hyperactivity disorder (ADHD), and tic disorders as some of the most common [46].

### Executive function and the cortico-striato-thalamo-cortical model of OCD

The cortico-striato-thalamo-cortical (CSTC) model of OCD [6, 31, 41], implicates executive function (EF) deficits and imbalances within CSTC brain circuits in OCD pathophysiology [22]. EF describes a set of interrelated, separable cognitive processes responsible for goal-directed behavior [32]. The exact hierarchy of functions continues to be debated but typically comprises specific subdomains, e.g., flexibility, working memory, and response inhibition [12]. The processes manifest behaviorally and are typically measured with neuropsychological tasks (targeting EF capacity in specific tasks) and questionnaires, such as the Behavior Rating Inventory of Executive Function (BRIEF) [14–16], targeting everyday EF. Questionnaires and task results are generally weakly correlated, indicating an issue related to validity or that they represent distinct, complementary EF expressions [47].

### Executive function pre-treatment and association with OCD symptom severity

Atypical EF is well documented in adults with OCD [3, 48] and to some extent in children (Abramovitch et al., 2015) [28] (Marzuki et al., 2020) [49], but associations with OCD symptom severity are inconsistent [1, 13, 35, 43, 49]. Questionnaire studies report more EF difficulties in children with OCD compared to controls, though not linked to symptom severity [24, 35, 43, 55]. To our knowledge, self-ratings in pediatric OCD have not been studied.

### Psychotherapy for pediatric OCD

Family-based cognitive-behavioral therapy (CBT) with exposure and response prevention (ERP) is an effective psychological intervention and first-line treatment for OCD [8, 34, 40, 50]. The child practices setting goals and achieving them through cognitive restructuring and behavior modification [39]. While effective for many, up to 40% of children are non- or partial responders to CBT [10, 11, 37, 39]. The mechanisms underlying therapeutic effects need to be further identified and targeted to improve outcomes. Relaxation training (RT), often used as a control intervention and sometimes combined with psychoeducation [10, 11, 39, 42], is less effective but still reduces symptoms [8, 37]. In contrast to CBT, RT does not involve ERP, which could be viewed as EF-training due to the active skill building training cognitive and behavioral modification.

### Executive function change after treatment and association with symptom change

Successful treatment of OCD is suggested to reduce EF difficulties, indicating EF as state-dependent [20]. Preliminary evidence suggests that test results of response inhibition, planning, and flexibility as well as BRIEF total score (General Executive Composite; GEC) improve after OCD treatment [4, 19, 20] but associations with symptom alleviation are unclear.

### Executive function as a moderator

EF deficits may also predict or moderate treatment response so that successful response to psychotherapy may depend on the individual’s pre-treatment EF [9, 20, 28]. Note that while a conceptual distinction can be made between predictors (main effects) or moderators (interaction with treatment type), we use the term “moderator” throughout for simplicity. In two studies, children with better parent-rated emotional control had less severe symptoms following CBT than children with poorer emotional control [29, 30] while others did not find an effect [20, 24, 43]. Most prior work was open-label and included medicated patients, potentially masking a moderation effect on psychotherapy.

In summary, as recently noted [22], longitudinal investigations of EF in pediatric OCD that include well-defined treatment- and control groups are needed.

### Aims

The current study is the first to examine EF in pediatric OCD via the BRIEF-2 [16] through self- and parent-ratings. We use data from the Danish TECTO (Treatment Effects of Family-Based Cognitive Therapy in Children and Adolescents with Obsessive Compulsive Disorder) randomized clinical trial (RCT), comparing effects of family-based CBT versus family-based psychoeducation and relaxation training (PRT), and data from a group of non-psychiatric control children (hereafter: controls) [37, 38].

Our aims were to examine the extent to which (1) EF difficulties are reported pre-treatment in patients relative to controls and whether EF difficulties in patients are associated with higher OCD symptom severity before treatment; (2) Psychological treatment is associated with alleviating EF difficulties in patients and whether this is associated with OCD symptom severity reduction; and (3) Pre-treatment EF in patients moderates treatment-related OCD symptom reduction. For Aims 2 and 3, analyses also examined differential effects for the two treatments (CBT versus PRT).

Our hypotheses were: Aim 1: (1.1) Greater EF difficulties will be reported in children with OCD pre-treatment compared with controls and (1.2) in patients greater EF difficulties will be associated with more severe OCD symptoms. Aim 2: (2.1) EF difficulties in children with OCD improve with psychotherapy, which is moderated by psychotherapy type with CBT outperforming PRT, and (2.2) Greater EF improvement will be associated with larger reductions in OCD symptoms. Aim 3: Pre-treatment EF difficulties moderate OCD symptom reduction after treatment. While our hypotheses were directional, we used two-sided tests to provide a more conservative evaluation of the effects.

## Methods

### Trial design

The study included participants from the Danish TECTO trial, which assesses benefits and harms of family-based CBT compared with an active control treatment (Family-Based Psychoeducation and Relaxation Training; PRT) in a superiority RCT design (ClinicalTrials.gov: NCT03595098). For a thorough description of study design and procedures, please see Olsen M.H. et al. [36]; Pagsberg et al. [38].

TECTO included 130 children and adolescents with OCD (age 8–17 years) and 90 control children between July 2018 and July 2022. Controls were recruited via the Danish Civil Registration System by identifying individuals with the same age and sex as an included patient. Although one-to-one matching was intended, this was not fully achieved due to limited control availability. Diagnostic status (for patients and controls) according to the International Classification of Diseases 10th Revision (ICD-10) [54] was based on transference of item criteria from assessments by the Kiddie Schedule for Affective Disorders and Schizophrenia – Present and Lifetime version (K-SADS-PL) [23]. Inclusion in the OCD group required a Children’s Yale-Brown Obsessive-Compulsive Scale (CY-BOCS) [45] total score of ≥ 16. Participants were excluded if they had an Intelligence Quotient (IQ) < 70 measured with age-appropriate Wechsler Scales [52, 53]. Patients were recruited from a single center in the Capital Region of Denmark and randomized to either family-based CBT or family-based PRT, both including 14 therapy sessions of 75 min each during a period of 16 weeks. Randomization was stratified by age (8–12 and 13–17 years) and CY-BOCS total score before treatment (moderate: 16–23 and severe: 24–40). Controls were recruited via the Danish national register. Patients were assessed before the first therapy session (baseline; week 0) and after the last therapy session (end-of-treatment; week 16), and the control children were assessed with the same time interval.

### Measures

#### OCD symptom severity

OCD symptom severity was assessed pre- and post-treatment with the clinician-rated semi-structured interview CY-BOCS total score. This score reflects frequency, impact, distress, resistance, and control of obsessions and compulsions on a scale from 0 to 40. The CY-BOCS was administered to the child (in most cases accompanied by their parent) by a trained clinician (medical doctor, psychologist, or psychology student under supervision).

#### Parent- and self-rated executive function

EF of the children was assessed with the BRIEF-2 questionnaires [15, 16] – both the parent-rated version (63 items) and the self-rated version (55 items – only children aged 11–18 years). The BRIEF-2 items describe problematic behavior related to EF-demanding tasks in everyday life and are rated according to frequency (“Never”, “Sometimes”, or “Often”). The questionnaire produces nine subscale scores for parent-ratings and seven for self-ratings. The scales add up to three index scores as well as a total score (GEC), reflecting the total amount of EF-related problematic behavior (for details, see Supplementary Table S1).

In the current study, we were interested in examining EF subdomains. Thus, BRIEF-2 subscale (hereafter: subdomains) scores were included in our analyses but not BRIEF index scores or GEC. If an informant was missing two or more items on a given subdomain, that scale score was excluded for that informant, while remaining scale scores were retained, as recommended by Gioia et al. [16]. Scale results were standardized to T-scores with a linear transformation, based on normative samples with a mean of 50 and a standard deviation of 10, using the Hogrefe Test System (HTS 5) [16]. Higher scores indicate higher levels of EF difficulties. The BRIEF-2 scales have demonstrated good psychometric properties. Internal consistency is acceptable to high (Cronbach’s *α* = 0.70–0.95) for all but two scales (initiating and self-monitoring) in Danish normative pediatric samples (age: 5–18 years) [16].

### Participants

We aimed to include all participants from the TECTO trial in the present study. We also included a control group to compare the OCD patients to a non-psychiatric group and to control for potential effects of repeated testing. Participants were included if at least one informant (child or parent) had reported BRIEF-2 at baseline. Thus, 114 children and adolescents (hereafter: children) with OCD and 74 control children were included in this study (for a full participant flow chart, see Supplementary Figures S1 and S2).

### Data analysis

Primary and secondary analyses are described in this section. Additional details of the statistical analysis plan are provided in the supplementary material. Following the Benjamini-Hochberg correction procedure, we used critical values of 0.01281 for parent-ratings and 0.00567 for self-ratings (details are provided in the supplementary material).

#### Executive function pre-treatment and associations with symptom severity (Aim 1)

Aim 1.1: Group differences (OCD versus controls) in EF domains (BRIEF-2 subdomains) before treatment were analyzed by two multivariate analyses of variance (MANOVAs) reporting Pillai’s Trace statistics – one for parent-report and one for self-report BRIEF data. In case of significant results, follow-up univariate analyses of variance (ANOVAs) were performed on each individual subdomain. To test the robustness of the group comparisons, sensitivity analyses were conducted by (1) including IQ and parental education as covariates, (2) restricting the OCD sample to patients without any comorbidity, and (3) restricting the sample to patients without specific comorbidity (Asperger’s, ADHD, generalized anxiety (GAD), and Tourette’s syndrome).

Aim 1.2: Associations between executive function and symptom severity were examined via two multiple linear regression analyses calculated to predict symptom severity (CY-BOCS Total Score) in OCD patients, based on the BRIEF subdomains as potential predictors.

Post-hoc descriptive analyses reported, for each subdomain, the proportion of participants falling within clinically elevated categories: T scores ≥ 60 = Slightly elevated, ≥ 65 = Potentially elevated, and ≥ 70 = Clinically elevated [16].

#### Executive function change after treatment and association with symptom change (Aim 2)

Aim 2.1: Change in EF during therapy was analyzed using two 2 (treatment group) × 2 (time) × 7 (BRIEF-2 subdomains) repeated measures analyses of variance (ANOVA) with BRIEF-2 T-Scores as the dependent variable. One ANOVA examined parent-rated data (note the parent-rated data were analyzed via 2 × 2 × 9 ANOVA as two more subdomains are determined from the parent data). The second ANOVA examined child self-rated data. Follow-up analyses report estimated marginal means (using the *emmeans* package in R) [27] pre- and post-treatment for BRIEF-2 subdomains in the two treatment groups and the control group.

Aim 2.2: We examined whether change in OCD symptom severity was associated with change in EF scores via two multiple linear regression analyses – one for parent-ratings and one for child-ratings. The models predicted post-treatment symptom severity (CY-BOCS Total Score) in OCD patients, based on the change in BRIEF-2 subdomains and pre-treatment CY-BOCS as potential predictors.

Additional ANOVAs were conducted to determine whether any time related reductions in symptom severity reflected practice and/or developmental effects (i.e., whether improvement was particularly marked in patients relative to controls). We conducted a 2 (group) × 2 (time) × 7 (BRIEF-2 subdomain) repeated measures ANOVA for self-ratings and a similar 2 × 2 × 9 ANOVA for parent-ratings.

#### Executive function as a moderator (Aim 3)

Aim 3: We used a Baron and Kenny moderation analysis [5] to examine if any BRIEF-2 subdomain pre-treatment moderated the relationship between treatment group and post-treatment symptom severity. We conducted two multiple linear regression analyses, calculated to predict post-treatment symptom severity (CY-BOCS). Fixed effects included pre-treatment CY-BOCS scores, treatment group (CBT versus PRT), all pre-treatment BRIEF-2 subdomains, and interaction terms between BRIEF-2 subdomain and treatment group. If the interaction term between the putative moderator and treatment group is significant, there is a moderation effect [21].

BRIEF-2 subdomains, treatment group, and CY-BOCS pre-treatment were mean-centered to reduce potential multicollinearity between the predictors and their interaction terms [21]. Thus, treatment groups (with almost the same N) were coded approximately as follows: PRT = −1 and CBT = 1.

## Results

Mean time between the two BRIEF-2 assessments for parent-ratings was 17.6 weeks (13.0–31.4.0.4 range) in the OCD group and 13.3 weeks (12.0–22.6.0.6) in the control group. For child self-ratings, mean time between assessments was 17.4 weeks (range: 13.0–31.4.0.4) in the OCD group and 16.0 weeks (range: 12.0–19.6.0.6) in the control group.

### Demographic and clinical characteristics

Demographic and clinical characteristics pre-treatment are presented in Table 1 for participants with complete data (similar numbers for patients with complete data at both time points; see Supplementary Table S2). Results are reported separately for parent- and self-rating samples, as these differ. Patients differed significantly from controls in parental education and IQ (see Table 1). The treatment groups did not differ on any measured characteristic.

**Table 1:** Demographic and Clinical Characteristics pre-treatment (Parent- and Self-Rating Samples)

	OCD Mean(SD), *n*(%)	CTR Mean(SD), *n*(%)	OCD vs. CTR *p*; Effect size		CBT Mean(SD), *n*(%)	PRT Mean(SD), *n*(%)	CBT vs. PRT *p*; Effect size	
Number of participants								
Parent-rating Self-ratings	112 82	74 52			54 43	58 39		
Number of females								
Parent-ratings Self-ratings	59 (52.7) 49 (59.8)	37 (50.0) 31 (59.6)	0.835 1	0.020 0.001	33.0 (61.1) 27.0 (62.8)	26 (44.8) 22 (56.4)	0.125 0.716	0.163 0.065
Age, years								
Parent-ratings Self-ratings	13.3 (2.88) 14.7 (1.99)	13.3 (2.74) 14.8 (1.92)	0.985 0.775	0.003 0.050	13.1 (2.94) 14.4 (2.06)	13.5 (2.82) 15.0 (1.90)	0.481 0.201	0.134 0.284
Parental education, years								
Parent-ratings Self-ratings	15.6 (2.11) 15.5 (2.19)	16.7 (1.83) 16.8 (1.57)	**< 0.001** **< 0.001**	0.569 0.675	15.5 (2.2) 15.3 (2.5)	15.7 (1.77) 15.7 (1.80)	0.607 0.443	0.134 174
Intelligence Quotient (IQ)								
Parent-ratings Self-ratings	99.2 (12.7) 98.9 (12.2)	105 (14.2) 104 (14.0)	**< 0.001** **< 0.039**	0.454 0.385	98.9 (12.0) 98.2 (11.8)	99.4 (13.5) 99.5 (12.8)	0.850 0.648	0.036 0.102
CY-BOCS Total Score								
Parent-ratings Self-ratings	25.2 (6.67) 25.2 (4.53)	- -	- -	- -	25.1 (4.6) 25.1 (4.6)	25.8 (4.73) 25.4 (4.48)	0.385 736	0.165 0.075
Comorbid disorders								
Asperger’s (F84.5)								
Parent-ratings Self-ratings	15 (13.4) 9 (11.0)	0 (0) 0 (0)	- -	- -	3 (8.11) 2 (7.14)	6 (18.75) 4 (19.05)	0.129 0.116	0.170 0.212
ADHD (F90.0)								
Parent-ratings Self-ratings	12 (10.7) 5 (6.1)	0 (0) 0 (0)	- -	- -	5 (13.51) 2 (7.14)	4 (12.50) 1 (4.67)	0.861 1	0.045 0.039
GAD (F41.1 & F93.8)								
Parent-ratings Self-ratings	9 (8.0) 7 (8.5)	0 (0) 0 (0)	- -	- -	1 (2.70) 0 (0)	4 (12.50) 3 (14.29)	0.201 0.086	0.154 0.233
Tourette’s (F95.2)								
Parent-ratings Self-ratings	7 (6.3) 4 (4.9)	0 (0) 0 (0)	- -	- -	2 (5.41) 1 (3.57)	2 (6.25) 2 (9.52)	0.494 1	0.102 0.011
Any comorbid disorder								
Parent-ratings Self-ratings	64 (57.1) 41 (50.0)	0 (0) 0 (0)	- -	- -	20 (54.05) 13 (46.43)	18 (56.25) 11 (52.38)	0.201 0.086	0.154 0.233

OCD = Patients with OCD. CTR = Control children. CBT = Patients who received cognitive-behavioral therapy (CBT). PRT = Patients who received psychoeducation and relaxation training (PRT). Effect size: As effect sizes we used Cohens *d* for numerical data and Cramer’s *V* for categorical data. CY-BOCS = Children’s Yale-Brown Obsessive-Compulsive Scale. Comorbid diagnosis is reported if present in > 3% of patients (self-ratings/parent-ratings). ADHD = Attention-Deficit/Hyperactivity Disorder. GAD = Generalized Anxiety Disorder

### Executive function pre-treatment and associations with symptom severity (Aim 1)

**Fig. 1:**
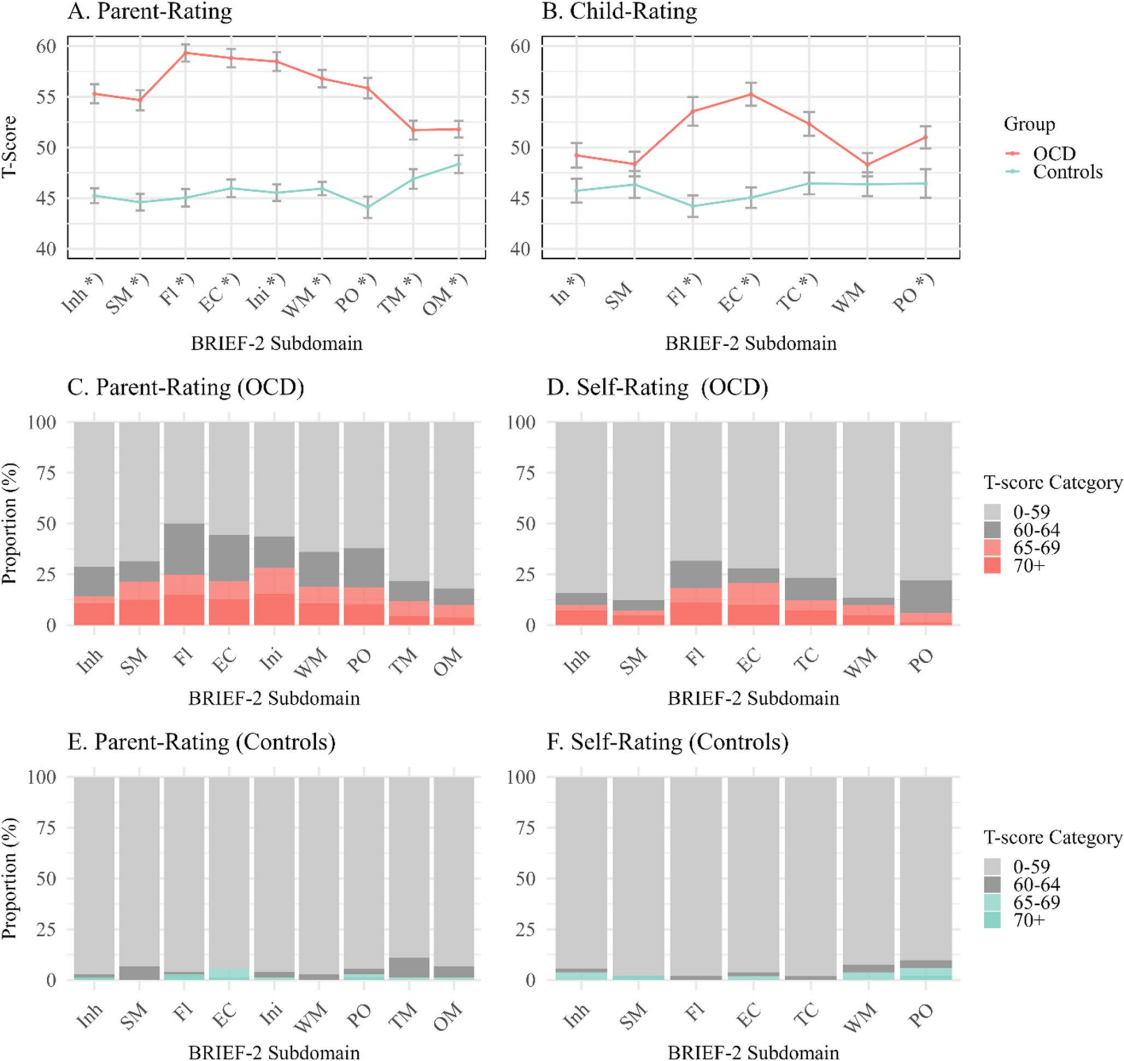
BRIEF-2 Domains Pre-Treatment. Note: BRIEF-2 = Behavior Rating Inventory of Executive Function, Second Edition [15]. Inh = Inhibition, SM = Self-Monitoring, Fl = Flexibility, EC = Emotional Control, Ini = Initiating, WM = Working Memory, PO = Planning/Organizing, TM = Task Monitoring, OM = Organization of Materials, TC = Task Completion

Aim 1.1: The MANOVA analyses indicated a significant multivariate effect of group (OCD versus controls) on the combined BRIEF-2 subdomains for both parent-ratings (Pillai’s Trace = 0.516, *F*(9,173) = 20.477, *p* <.001, ${{\eta}_{p}}^{2}$ = 0.50) and self-ratings (Pillai’s Trace = 0.314, *F*(7,126) = 8.235, *p* <.001, ${{\eta}_{p}}^{2}$ = 0.31). Given the significant main effects of group, follow-up ANOVAs were performed to investigate the effect on each BRIEF-2 subdomain individually. Parent-rated data indicated a significant effect of group on all domains, and child self-rated data revealed group effects for all but self-monitoring and working memory (Fig. 1A and B; for more details, see Supplementary Tables S3 and S4). This pattern was robust across all sensitivity analyses (for more details, see Supplementary Tables S5-S7). Notably, while all patient mean scores fall within the normal range (up to T = 60) [16] and control mean scores fall a bit below the normative mean of T = 50, a considerable proportion of patients, but not controls, fall above the normal range within each subdomain [16] (Fig. 1C and F; for more details, see Supplementary Tables S8 and S9). Additionally, patients are more likely than controls to fall above the normal range on one or more subdomains (Supplementary Table S10; Figures S7-9).

Aim 1.2: The multiple regression analyses (predicting symptom severity (CY-BOCS total score) in patients, based on BRIEF-2 subdomain) revealed a significant regression model for the self-ratings (*F*(7,74) = 3.904, *p* =.001, *R*^*2*^ = 0.201) but not for parent-ratings (*F*(9,100) = 2.026, *p* =.044, *R*^*2*^ = 0.078) data.

The self-rating model accounted for 20.1% of the variance, and examination of the individual predictors indicated that pre-treatment flexibility was a significant predictor of pre-treatment CY-BOCS score (β = 0.21, *t*(74) = 4.272, *p* <.001) whereas the rest of the subdomains were not (see Supplementary Material, Table S11). Thus, patients who had a higher flexibility score on BRIEF-2 (i.e., more flexibility problems) showed a higher CY-BOCS total score at baseline (i.e., more severe OCD symptoms) than patients with lower flexibility scores.

### Executive function change after treatment and association with symptom change (Aim 2)

**Fig. 2:**
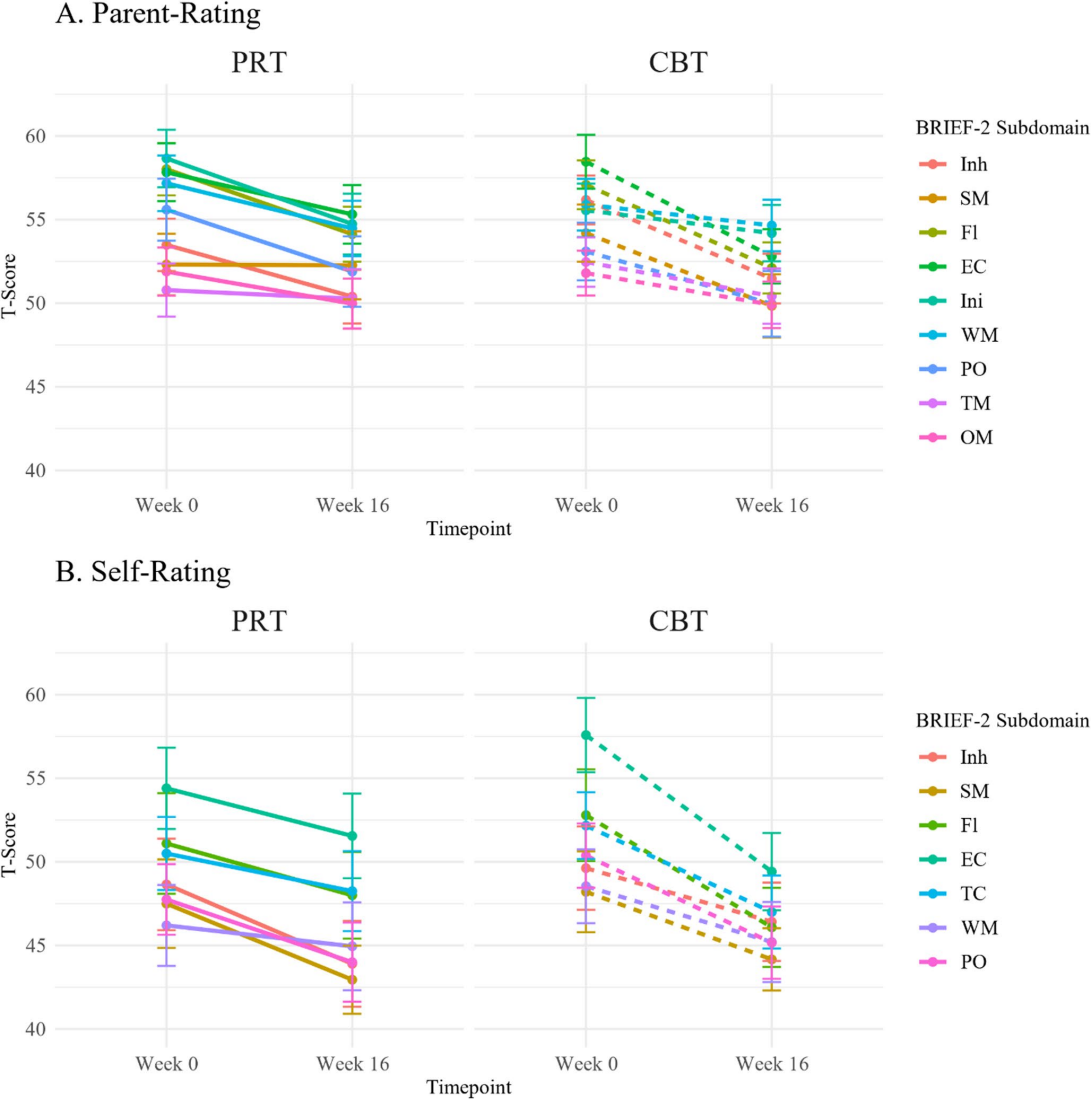
BRIEF-2 Domains Pre- and Post-Treatment by Treatment Group. Note: PRT = Patients who received psychoeducation and relaxation training (PRT). CBT = Patients who received cognitive-behavioral therapy (CBT). BRIEF-2 = Behavior Rating Inventory of Executive Function, Second Edition [15]. Inh = Inhibition, SM = Self-Monitoring, Fl = Flexibility, EC = Emotional Control, Ini = Initiating, WM = Working Memory, PO = Planning/Organizing, TM = Task Monitoring, OM = Organization of Materials, TC = Task Completion

Aim 2.1: The 2 (treatment group) × 2 (time) × 9 (BRIEF-2 subdomain) repeated measures ANOVA for parent-rated BRIEF-2 T-scores revealed significant main effects of time (*F*(1, 67) = 25.336; *p* <.001; ${\eta}_{p}^{2}$ = 0.27) and of BRIEF-2 subdomain (*F*(5, 334.79) = 8.313; *p* <.001; ${\eta}_{p}^{2}$ = 0.11). The same was seen for the 2 × 2 × 7 repeated measures ANOVA for self-rated BRIEF-2 T-scores (*F*(1, 43) = 21.762; *p* <.001; ${\eta}_{p}^{2}$ = 0.34); *F*(4.56, 195.96) = 7.39; *p* <.001; ${\eta}_{p}^{2}$ = 0.15). Thus, both parents and children reported EF improvements after treatment, and analyses showed significant differences in the T-scores of different BRIEF-2 subdomains (see Fig. 2).

Follow-up analyses stated that, for parent-ratings, the time × BRIEF-2 subdomain interaction and the treatment group × time × BRIEF-2 subdomain interaction were significant (*F*(6.57, 439.95) = 2.596; *p* =.014; ${\eta}_{p}^{2}$ = 0.04, and (*F*(6.57, 439.95) = 2.452; *p* =.020; ${\eta}_{p}^{2}$ = 0.04, respectively). Thus, the treatment groups differed in improvement levels in EF across BRIEF-2 subdomains (for more details, see Supplementary Figure S10).

No other main effects or interactions were significant (for full details, see Supplementary Tables S12 and S13).

Follow-up analyses showed a significant treatment × time interaction for self-monitoring (*F*(1,67) = 6.08, *p* =.010), where CBT demonstrated a notable improvement in self-monitoring (*t*(67) = 3.661, *p* <.001) relative to PRT (*t*(67) = 0.037, *p* =.971).

Follow-up analyses estimating pre- and post-treatment scores for all BRIEF-2 domains in the two treatment groups as well as in the control group are shown in Supplementary Tables S14-S17. EF remained stable in the control group while it improved in the OCD group (in both treatments) during the assessment period.

Post-hoc descriptive analyses reported the proportion of patients that fall within the clinically elevated categories (see Supplementary Figure S11).

Aim 2.2: The multiple linear regression analyses predicting change in symptom severity (CY-BOCS Total Score) from change in BRIEF-2 subdomains were not significant for neither parent- nor child rated data (*F*(9,59) = 0.978, *p* =.467; *F*(7,40) = 0.751, *p* =.631). Thus, symptom improvement was not predicted by change in executive function.

### Executive function as a moderator (Aim 3)

Aim 3: The multiple regression analyses of CY-BOCS post-treatment (higher means more severe symptoms) revealed significant regression models for both parent-ratings (*F*(20,77) = 2.191, *p* =.008, *R*^*2*^ = 0.363) and self-ratings (*F*(16,57) = 2.578, *p* =.0045, *R*^*2*^ = 0.420). Thus, pre-treatment EF difficulties and treatment type explain a significant amount of variance in post-treatment symptom severity. Full regression results, including all predictors and interactions, are available in Supplementary Tables S18 and S19.

The parent-rating model accounted for 36.3% of the variance. Follow-up analyses showed that pre-treatment working memory was a significant predictor (*β* = −0.60, *t*(85) = −3.189, *p* =.002), while there was no interaction with treatment group; children with more working memory pre-treatment difficulties showed greater benefits from psychotherapy (whether CBT or PRT). There was a significant interaction between treatment group and emotional control (*β* = −0.70, *t*(85) = −2.509, *p* =.014); emotional control moderates the effect of treatment on OCD so that more pre-treatment emotional control difficulties were associated with less severe OCD symptoms following treatment with CBT but not PRT (due to the coding of treatment allocation; CBT ≈ 1 and PRT ≈ −1, a negative coefficient reflects a steeper negative slope for CBT).

The self-rating model accounted for 42.0% of the variance in CY-BOCS post-treatment. Follow-up analyses showed that pre-treatment task-completion and working memory were significant predictors (*β* = 0.41, *t*(57) = 3.126, *p* =.003; β = −0.35, *t*(57) = −2.296, *p* =.025). Children reporting working memory pre-treatment difficulties showed greater benefits from psychotherapy (CBT or PRT), while children reporting greater pre-treatment task completion difficulties showed less benefit from treatment. Like for parent-ratings, there was a significant interaction between treatment group and emotional control (*β* = −0.66, *t*(57) = −2.644, *p* =.011) as well as between treatment group and task completion (*β* = 0.53, *t*(57) = 2.056, *p* =.044). Pre-treatment emotional control difficulties were associated with less severe OCD symptoms following CBT rather than PRT.

See Supplementary Figures S12-S17 for plots of the unique predictor effects, controlled for other variables in the models, including interaction plots.

## Discussion

This study examined EF in pediatric OCD, focusing on pre-treatment difficulties, treatment-related change, and moderation of psychotherapy outcomes. Compared to controls, children with OCD showed EF difficulties at baseline, particularly in flexibility and emotional control, which improved following both CBT and PRT. EF improvements in patients after 16 weeks of psychotherapy were not associated with symptom reduction, but pre-treatment EF moderated treatment outcomes in distinct ways, highlighting potential value in tailoring interventions.

### Executive function pre-treatment and associations with symptom severity (Aim 1)

EF skills are crucial for adaptive behavior in everyday life. In OCD, EF impairment is suggested to underlie difficulties in controlling obsessive thoughts and compulsive behavior [22]. Consistent with prior studies [20, 35, 43, 55], children with OCD in our study displayed EF difficulties relative to controls, particularly in flexibility, emotional control, and task completion. Self-rated flexibility was modestly associated with OCD symptom severity, aligning partially with adult data and suggesting a potential role for flexibility in OCD pathology [1]. However, it should be noted that most previous work with pediatric and adult samples with OCD has found little evidence for such an association [13, 35, 43]. Indeed, in our previous work with this sample using neuropsychological tests, underperformance in EF was also observed in OCD-patients but not associated with severity of OCD symptoms [49].

It should be noted that EF is increasingly conceptualized as a transdiagnostic factor implicated across psychiatric disorders [22, 41, 44]. Accordingly, the EF alterations observed here warrant cautious interpretation as disorder-specific, particularly given the high rates of psychiatric comorbidity. Sensitivity analyses suggested that the findings were generally robust, but the possibility that psychopathology other than OCD contributed to the observed patterns cannot be fully excluded.

### Executive function change after treatment and association with symptom change (Aim 2)

Prior, albeit limited, studies have examined the impact of psychotherapy for pediatric OCD on EF. Neuropsychological work has indicated that measures of inhibition, flexibility, and planning improved from pre- to post treatment [4, 19]. Additionally, parent-rated BRIEF Global Executive Composite was reported to improve after CBT [20]. Our results are broadly consistent with this previous literature; parents of patients reported improvement in all BRIEF-2 subdomains following psychotherapy (whether CBT or PRT), and the patients themselves reported improvement in several subdomains (whether CBT or PRT). Importantly, we extend this literature in several ways. First, by incorporating individual BRIEF-2 subdomains into our statistical model, we could determine that most domains improve as a function of psychotherapy (whether CBT or PRT). This is seen whether EF is indexed via parent- or self-ratings. Notably, EF in the control group did not significantly change over the treatment interval whether indexed by parent- or self-ratings (which was also seen in the American norm material [15]; i.e., the results cannot be attributed to repeated testing (see Supplementary Tables S13 and S14). Second, parents reported differing improvement levels across subdomains, with the largest improvement seen for flexibility and emotional control as well as inhibition and planning/organizing. It should be noted that the largest effect sizes for self-rated data were also seen within these subdomains. As such, our results are consistent with previous work reporting improvement in both children [4, 19] and adults [26, 33, 51] reporting improvement. Third, we observed a significant treatment group × time × BRIEF-2 subdomain interaction for the parent rated data, i.e., there were significantly different improvement levels of BRIEF-2 subdomains as a function of treatment group. Follow-up results revealed that this particularly reflected a notable improvement in self-monitoring following CBT, while no improvement was seen for PRT on this subdomain.

The self-monitoring domain measures the ability to evaluate own behaviors/skills and how these affect other people [16]. It is conceivable that CBT has a greater impact on self-monitoring than PRT due to the ERP component, which trains awareness of thinking and behavior, and how to adjust them. However, given the exploratory nature of our follow up tests, this result should be treated with caution.

Several features should be noted with respect to the large improvement in flexibility, emotional control, inhibition, and planning/organizing subdomains. Both flexibility and inhibition difficulties are putative key features of OCD that might improve with treatment [22]. It is suggested that if patients train their ability to inhibit unwanted thoughts/behavior and flexibly update this with appropriate alternate solutions during CBT, this might mediate reduction in OCD symptoms. Two pediatric studies in OCD have identified improvement of neuropsychological test-performance on flexibility, inhibition, and planning tasks after CBT [4, 19], and some adult studies found the same for flexibility [26, 33, 51]. Our study supports inhibition, flexibility, and planning/organizing improving after psychotherapy for OCD, but opposite to our expectations, this effect was not specific to CBT but could also be obtained through PRT. Moreover, both parents and children recognized significant improvement of the emotional control domain in our study. One aspect of emotional control, temper outburst, is observed to improve with CBT for pediatric OCD [25]. The current study supports improvement of emotional control during psychotherapy but did not see a specific effect for CBT. Notably, whereas the 8-item BRIEF-2 scale focuses on strong behavioral reactions [16], the 36-item DERS might better capture psychological aspects of emotional control (such as acceptance and awareness of one’s own emotions), which could explain the difference.

Change in EF following psychotherapy was not associated with the magnitude of symptom alleviation in OCD symptomatology. While inconsistent with our predictions, this result is in line with a previous study using parent-rated BRIEF scores [20]. In short, while psychotherapy improves EF in patients with OCD as well as alleviating their symptoms (especially CBT; [37]), these impacts appear relatively independent of each other. These data suggest that while many patients with OCD experience EF-related difficulties that improve with psychotherapy, these problems may be secondary to the core pathophysiology driving OCD symptoms. Improvement in EF, however, may still enhance daily functioning and well-being, reflecting benefits beyond the trajectory of OCD symptom reduction.

### Executive function as a moderator (Aim 3)

Our study extends prior literature [30] by adding self-ratings and comparing non-medicated children in a parallel psychotherapy design, as suggested by Jalal et al. [22]. Our results suggest that several EF domains predict symptom severity post-treatment and differentiate CBT and PRT in distinct ways. Children with emotional control difficulties pre-treatment benefitted relatively more from CBT than PRT. This may suggest that children with emotional control difficulties pre-treatment may benefit relatively more from CBT compared to PRT, possibly because CBT with ERP helps children build skills for identifying and managing strong emotions, while PRT provides less support for handling such distress. Pre-treatment working memory difficulties predict less severe OCD symptoms following psychotherapy when looking at all patients (whether CBT or PRT). Thus, pre-treatment working memory difficulties may allow patients to benefit relatively more from treatment, and children with such difficulties seem to respond well to the intensive support and structure provided in psychotherapy. Finally, more difficulties with task-completion were associated with more severe symptoms after treatment for all patients (regardless CBT or PRT). Children with task-completion problems struggle to complete tasks within a time frame or at a sufficient pace, and it is possible they also struggle to do the tasks (both in session and as homework) required for psychotherapy. Altogether, children with task-completion difficulties pre-treatment may require additional support to complete therapeutic tasks.

### Strengths and limitations

Our study has numerous strengths. We provide one of the most complex descriptions of daily-life EF in pediatric OCD to date by using a multiple-informant approach, assessing multiple EF domains before and after two types of treatment, and compare a non-medicated sample to a thoroughly screened control group. Reporting specific EF domains allows our findings to be compared with the neuropsychological literature, which often focuses on individual EFs rather than overall capacity. The repeated measures in the control group demonstrate stability of EF over time, supporting the view that observed changes in patients are related to therapy effects. Finally, by using the BRIEF-2, we assessed EF in a real-life context, in contrast to task-based measures that capture momentary performance in a specific setting and may not reflect overall daily functioning.

Several limitations should be considered. First, the assessment of EF using the BRIEF-2 relies on subjective reports. While such measures offer ecological validity, their modest correspondence with task-based EF measures and their susceptibility to rater bias limit inferences about underlying neurocognitive mechanisms. At the same time, performance-based EF tests have been criticized for limited reliability and sensitivity. Accordingly, we consider parent- and self-reported measures to be as relevant and informative as task-based assessments, particularly for capturing EF as it manifests in everyday functioning. Second, the sample size for self-rated data was smaller than for parent-rated, mainly due to the age restrictions (> 11 years), and some attrition which occurred before follow-up. While the study was powered a priori for the primary outcome of the TECTO trial (CY-BOCS) rather than BRIEF-2, several moderate effects were detected for BRIEF-2 outcomes indicating that the available sample size still allowed us to detect effects of at least this magnitude while smaller effects may have gone undetected. Third, our study could have potential selection-biases deriving from which parents/children completed the BRIEF-2. If completion were dependent on, for example, symptom severity, comorbidity, or another variable, this might impact the generalizability of our results. Notably, the considerable patient drop-out rate (which was higher for PRT) could introduce bias. Fourth, the high rate of psychiatric comorbidity in our sample, reflecting the typical pediatric OCD population, made it challenging to isolate effects specific to OCD. Nonetheless, our findings remained robust in sensitivity analyses. Finally, our control group had a higher mean IQ than the normative average and their parents had higher educational levels. While this is to some extent expected in children without mental illness [18], it is possible that children with higher mean IQ and parents with higher educational levels were more likely to participate in research, resulting in an artificially advantaged control group and potentially amplifying group differences. To provide a meaningful context, we reported T-scores and proportion of children above clinical thresholds (≥ 60, ≥ 65, ≥ 70) highlighting a substantial subgroup of children with OCD who have difficulties well beyond both controls and population norms.

## Conclusion

This study is the first to assess patient-reported EF in pediatric OCD and the first to report longitudinal assessment of parent- and self-rated EF across two psychotherapies in non-medicated youth with OCD compared with a non-psychiatric control group. While the BRIEF-2 scores were reported to be within normal limits for most children with OCD, a considerable subgroup showed pronounced EF difficulties, particularly in flexibility and emotional control, which improved after treatment with either CBT or PRT. EF change was not associated with symptom improvement, but these EF gains may nonetheless enhance daily functioning and quality of life. Pre-treatment EF moderated treatment outcomes in distinct ways, highlighting EF ratings as a potential tool in tailoring interventions. Specifically, children with emotional control difficulties may benefit relatively more from CBT, those with working memory difficulties appear to respond well to structured interventions, and children with task-completion problems may require additional therapeutic support to complete their tasks during psychotherapy effectively. This study establishes that child-reported EF difficulties exist in pediatric OCD and, together with parent-report, reveal that pre-treatment EF profiles can provide valuable guidance for tailoring personalized interventions.

## Supplementary Information

Below is the link to the electronic supplementary material.

Supplementary Material 1 (DOCX 1.06 MB)

## Data Availability

Available upon request.

## Code availability

Available upon request.

## **Declarations**

### Trial registration number

ClinicalTrials.gov NCT03595098.

## References

[1] Abramovitch A, McCormack B, Brunner D, Johnson M, Wofford N (2019) The impact of symptom severity on cognitive function in obsessive-compulsive disorder: a meta-analysis. Clin Psychol Rev 67:36–44. 10.1016/j.cpr.2018.09.003 30528984 10.1016/j.cpr.2018.09.003

[2] Abramowitz JS, Taylor S, McKay D (2009) Obsessive-compulsive disorder. Lancet 374(9688):491–499. 10.1016/S0140-6736(09)60240-3 19665647 10.1016/S0140-6736(09)60240-3

[3] Abreu D, Almeida N, Santos N (2024) Executive functions’ alterations in obsessive-compulsive disorder: a systematic literature review. Psicol Argumento 42(117):117. 10.7213/psicolargum.42.117.AO15

[4] Andrés S, Lázaro L, Salamero M, Boget T, Penadés R, Castro-Fornieles J (2008) Changes in cognitive dysfunction in children and adolescents with obsessive-compulsive disorder after treatment. J Psychiatr Res 42(6):507–514. 10.1016/j.jpsychires.2007.04.004 17599358 10.1016/j.jpsychires.2007.04.004

[5] Baron RM, Kenny DA (1986) The moderator–mediator variable distinction in social psychological research: conceptual, strategic, and statistical considerations. J Pers Soc Psychol 51(6):1173–1182. 10.1037/0022-3514.51.6.1173 3806354 10.1037//0022-3514.51.6.1173

[6] Brem S, Hauser TU, Iannaccone R, Brandeis D, Drechsler R, Walitza S (2012) Neuroimaging of cognitive brain function in paediatric obsessive compulsive disorder: A review of literature and preliminary meta-analysis. J Neural Transm 119(11):1425–1448. ttps://doi.org/10.1007/s00702-012-0813-z 22678698 10.1007/s00702-012-0813-z

[7] Cervin M (2023) Obsessive-Compulsive disorder: diagnosis, clinical features, nosology, and epidemiology. Psychiatr Clin North Am 46(1):1–16. 10.1016/j.psc.2022.10.006 36740346 10.1016/j.psc.2022.10.006

[8] Cervin M, McGuire JF, D’Souza JM, De Nadai AS, Aspvall K, Goodman WK, Andrén P, Schneider SC, Geller DA, Mataix-Cols D, Storch EA (2024) Efficacy and acceptability of cognitive‐behavioral therapy and serotonin reuptake inhibitors for pediatric obsessive‐compulsive disorder: a network meta‐analysis. J Child Psychol Psychiatry 65(5):594–609. 10.1111/jcpp.13934 38171647 10.1111/jcpp.13934

[9] Fitzgerald KD, Schroder HS, Marsh R (2021) Cognitive control in pediatric obsessive-compulsive and anxiety disorders: brain-behavioral targets for early intervention. Biol Psychiatry 89(7):697–706. 10.1016/j.biopsych.2020.11.012 33454049 10.1016/j.biopsych.2020.11.012 PMC8353584

[10] Freeman JB, Garcia AM, Coyne L, Ale C, Przeworski A, Himle M, Compton S, Leonard HL (2008) Early childhood OCD: preliminary findings from a family-based cognitive-behavioral approach. J Am Acad Child Adolesc Psychiatry 47(5):5. 10.1097/CHI.0b013e31816765f9 10.1097/CHI.0b013e31816765f9 PMC2820297 18356758

[11] Freeman JB, Sapyta J, Garcia A, Compton S, Khanna M, Flessner C, FitzGerald D, Mauro C, Dingfelder R, Benito K, Harrison J, Curry J, Foa E, March J, Moore P, Franklin M (2014) Family-based treatment of early childhood obsessive-compulsive disorder: the pediatric obsessive-compulsive disorder treatment study for young children (POTS Jr)—a randomized clinical trial. JAMA Psychiatr 71(6):689. 10.1001/jamapsychiatry.2014.170 10.1001/jamapsychiatry.2014.170 PMC4511269 24759852

[12] Friedman NP, Robbins TW (2022) The role of prefrontal cortex in cognitive control and executive function. Neuropsychopharmacology 47(1):72–89. 10.1038/s41386-021-01132-0 34408280 10.1038/s41386-021-01132-0 PMC8617292

[13] Geller DA, Abramovitch A, Mittelman A, Stark A, Ramsey K, Cooperman A, Baer L, Stewart SE (2018) Neurocognitive function in paediatric obsessive-compulsive disorder. World J Biol Psychiatry 19(2):142–151. 10.1080/15622975.2017.1282173 28090807 10.1080/15622975.2017.1282173 PMC5555842

[14] Gioia GA, Isquith PK, Guy SC, Kenworthy L (2000) Test review behavior rating inventory of executive function. Child Neuropsychol 6(3):235–238. 10.1076/chin.6.3.235.3152 11419452 10.1076/chin.6.3.235.3152

[15] Gioia GA, Isquith PK, Guy SC, Kenworthy L (2015) Behavior Rating Inventory of Executive Function^®^, Second Edition (BRIEF-II). Professional Manual. Psychological Assesment Resources (PAR) Inc

[16] Gioia GA, Isquith PK, Guy SC, Kenworthy L (2018) BRIEF: Behavior Rating Inventory of Executive Function, Second Edition (BRIEF-II). Vedledning (dansk udgave). Hogrefe Psykologisk Forlag

[17] Heyman I, Mataix-Cols D, Fineberg NA (2006) Obsessive-compulsive disorder. 333, 6 10.1136/bmj.333.7565.424 PMC1553525 16931840

[18] Holstein BE, Pant SW, Ammitzbøll J, Laursen B, Madsen KR, Skovgaard AM, Pedersen TP (2021) Parental education, parent–child relations and diagnosed mental disorders in childhood: prospective child cohort study. Eur J Public Health 31(3):514–520. 10.1093/eurpub/ckab053 33880520 10.1093/eurpub/ckab053

[19] Huyser C, Veltman DJ, Wolters LH, De Haan E, Boer F (2010) Functional magnetic resonance imaging during planning before and after cognitive-behavioral therapy in pediatric obsessive-compulsive disorder. J Am Acad Child Adolesc Psychiatry 49(12):1238-1248e.e5. 10.1016/j.jaac.2010.08.007 21093773 10.1016/j.jaac.2010.08.007

[20] Hybel KA, Mortensen EL, Lambek R, Højgaard DRMA, Thomsen PH (2017) Executive function predicts cognitive-behavioral therapy response in childhood obsessive-compulsive disorder. Behav Res Ther 99:11–18. 10.1016/j.brat.2017.08.009 28881220 10.1016/j.brat.2017.08.009

[21] Jaccard J, Turrisi R (2003) Interaction Effects in Multiple Regression, 2nd edn. SAGE 10.1207/s15327906mbr2504_4 26820822

[22] Jalal B, Chamberlain SR, Sahakian BJ (2023) Obsessive-compulsive disorder: etiology, neuropathology, and cognitive dysfunction. Brain Behav 13(6):e3000. 10.1002/brb3.3000 37137502 10.1002/brb3.3000 PMC10275553

[23] Kaufman J, Birmaher B, Brent D, Rao U, Flynn C, Moreci P, Williamson D, Ryan N (1997) Schedule for affective disorders and schizophrenia for school-age children-present and lifetime version (K-SADS-PL): initial reliability and validity data. J Am Acad Child Adolesc Psychiatry 36(7):980–988. 10.1097/00004583-199707000-00021 9204677 10.1097/00004583-199707000-00021

[24] Killion B, Marklin M, O’Connor E, Freeman JB, Cain GH, Walther M, Benito KG (2024) Executive Functioning, Family Accommodation, and Treatment Response in Youth with OCD and Comorbid ADHD in a Partial Hospital Program. Child Psychiatry Hum Dev. 10.1007/s10578-024-01792-1 39581890

[25] Krebs G, Bolhuis K, Heyman I, Mataix-Cols D, Turner C, Stringaris A (2013) Temper outbursts in paediatric obsessive‐compulsive disorder and their association with depressed mood and treatment outcome. J Child Psychol Psychiatry 54(3):313–322. 10.1111/j.1469-7610.2012.02605.x 22957831 10.1111/j.1469-7610.2012.02605.x PMC4026039

[26] Kuelz AK, Riemann D, Halsband U, Vielhaber K, Unterrainer J, Kordon A, Voderholzer U (2006) Neuropsychological impairment in obsessive-compulsive disorder—improvement over the course of cognitive behavioral treatment. J Clin Exp Neuropsychol 28(8):1273–1287. 10.1080/13803390500507246 17050258 10.1080/13803390500507246

[27] Lenth RV, Piaskowski J, Banfai B, Bolker B, Buerkner P, Giné-Vázquez I, Hervé M, Jung M, Love J, Miguez F, Riebl H, Singmann H (2025) *e*mmeans: Estimated Marginal Means, aka Least-Squares Means (Version 2.0.0) [Computer software]. https://cran.r-project.org/web/packages/emmeans/index.html

[28] López-Hernández P, Sánchez-Meca J, Rosa-Alcázar Á, Rosa-Alcázar AI (2022) A meta-analytic study on executive function performance in children/adolescents with OCD. An Psicol 38(3):478–488. 10.6018/analesps.305411

[29] McKenzie ML, Donovan CL, Mathieu SL, Hyland WJ, Farrell LJ (2020) Variability in emotion regulation in paediatric obsessive-compulsive disorder: associations with symptom presentation and response to treatment. J Obsessive Compuls Relat Disord 24:100502. 10.1016/j.jocrd.2019.100502

[30] McNamara JPH, Reid AM, Balkhi AM, Bussing R, Storch EA, Murphy TK, Graziano PA, Guzick AG, Geffken GR (2014) Self-regulation and other executive functions relationship to pediatric OCD severity and treatment outcome. J Psychopathol Behav Assess 36(3):432–442. 10.1007/s10862-014-9408-3

[31] Milad MR, Rauch SL (2012) Obsessive-compulsive disorder: beyond segregated cortico-striatal pathways. Trends Cogn Sci 16(1):43–51. 10.1016/j.tics.2011.11.003 22138231 10.1016/j.tics.2011.11.003 PMC4955838

[32] Miyake A, Friedman NP, Emerson MJ, Witzki AH, Howerter A, Wager TD (2000) The unity and diversity of executive functions and their contributions to complex “frontal lobe” tasks: a latent variable analysis. Cogn Psychol 41(1):49–100. 10.1006/cogp.1999.0734 10945922 10.1006/cogp.1999.0734

[33] Moritz S, Kloss M, Katenkamp B, Birkner C, Hand I (1999) *Neurocognitive functioning in OCD before and after treatment*. *241*(8)

[34] National Institute for Health and Care Excellence (NICE) (2005), November 29 *Obsessive-compulsive disorder and body dysmorphic disorder: Treatment | Guidance | NICE*. NICE. https://www.nice.org.uk/guidance/cg31 39480980

[35] Negreiros J, Best JR, Yamin DF, Belschner L, Lin S, Stewart SE (2020) Test-based versus parent ratings of executive function in pediatric Obsessive-Compulsive Disorder. J Obsessive Compuls Relat Disord 24:100495. 10.1016/j.jocrd.2019.100495

[36] Olsen MH, Hagstrom J, Lonfeldt NN, Uhre C, Uhre V, Pretzmann L, Christensen SH, Thoustrup C, Korsbjerg NLJ, Mora-Jensen A-R, Ritter M, Engstrom J, Lindschou J, Siebner HR, Verhulst F, Jeppesen P, Jepsen JRM, Vangkilde S, Thomsen PH, Jakobsen JC (2022) Family-based cognitive behavioural therapy versus family-based relaxation therapy for obsessive-compulsive disorder in children and adolescents (the TECTO trial): A statistical analysis plan for the randomised clinical trial. Trials 23(1):854. 10.1186/s13063-022-06799-4 36203215 10.1186/s13063-022-06799-4 PMC9535232

[37] Pagsberg AK, Lønfeldt NN, Thoustrup CL, Korsbjerg NLJ, Uhre CF, Christensen SH, Uhre VF, Mora-Jensen A-R, Ritter M, Pretzmann L, Ingstrup HK, Moltke BB, Harboe GS, Thorsen ED, Clemmensen LKH, Lindschou J, Engstrøm J, Gluud C, Siebner HR, Plessen KJ (2025) Family-based cognitive behavioral therapy versus family-based psychoeducation and relaxation training for obsessive-compulsive disorder in children and adolescents: A randomized clinical trial (TECTO). Eur Child Adolesc Psychiatry. 10.1007/s00787-025-02797-4 40742552 10.1007/s00787-025-02797-4 PMC12743107

[38] Pagsberg AK, Uhre C, Uhre V, Pretzmann L, Christensen SH, Thoustrup C, Clemmesen I, Gudmandsen AA, Korsbjerg NLJ, Mora-Jensen A-R, Ritter M, Thorsen ED, Halberg KSV, Bugge B, Staal N, Ingstrup HK, Moltke BB, Kloster AM, Zoega PJ, Plessen KJ (2022) Family-based cognitive behavioural therapy versus family-based relaxation therapy for obsessive-compulsive disorder in children and adolescents: Protocol for a randomised clinical trial (the TECTO trial). BMC Psychiatry 22(1):204. 10.1186/s12888-021-03669-2 35305587 10.1186/s12888-021-03669-2 PMC8933964

[39] Piacentini J, Bergman RL, Chang S, Langley A, Peris T, Wood JJ, McCracken J (2011) Controlled comparison of family cognitive behavioral therapy and psychoeducation/relaxation training for child obsessive-compulsive disorder. J Am Acad Child Adolesc Psychiatry 50(11):11. 10.1016/j.jaac.2011.08.003 10.1016/j.jaac.2011.08.003 PMC3205429 22024003

[40] Reid JE, Laws KR, Drummond L, Vismara M, Grancini B, Mpavaenda D, Fineberg NA (2021) Cognitive behavioural therapy with exposure and response prevention in the treatment of obsessive-compulsive disorder: A systematic review and meta-analysis of randomised controlled trials. Compr Psychiatry 106:152223. 10.1016/j.comppsych.2021.152223 33618297 10.1016/j.comppsych.2021.152223

[41] Robbins TW, Banca P, Belin D (2024) From compulsivity to compulsion: The neural basis of compulsive disorders. Nat Rev Neurosci 25(5):313–333. 10.1038/s41583-024-00807-z 38594324 10.1038/s41583-024-00807-z

[42] Russman Block S, Norman LJ, Zhang X, Mannella KA, Yang H, Angstadt M, Abelson JL, Himle JA, Taylor SF, Fitzgerald KD (2023) Resting-State Connectivity and Response to Psychotherapy Treatment in Adolescents and Adults With OCD: A Randomized Clinical Trial. Am J Psychiatry 180(1):89–99. 10.1176/appi.ajp.21111173 36475374 10.1176/appi.ajp.21111173 PMC10956516

[43] Rydqvist F, Hoff E, Daukantaitè D, Cervin M (2023) Everyday executive functioning in pediatric obsessive-compulsive disorder: Diagnostic specificity, clinical correlations, and outcome. BMC Psychiatry 23(1):622. 10.1186/s12888-023-05111-1 37620782 10.1186/s12888-023-05111-1 PMC10464101

[44] Sadozai AK, Sun C, Demetriou EA, Lampit A, Munro M, Perry N, Boulton KA, Guastella AJ (2024) Executive function in children with neurodevelopmental conditions: A systematic review and meta-analysis. Nat Hum Behav 8(12):2357–2366. 10.1038/s41562-024-02000-9 39424962 10.1038/s41562-024-02000-9 PMC11659155

[45] Scahill L, Riddle MA, McSWIGGIN-HARDIN M, Ort SI, King RA, Goodman WK, Cicchetti D, Leckman JF (1997) Children’s Yale-Brown Obsessive Compulsive Scale: Reliability and Validity. J Am Acad Child Adolesc Psychiatry 36(6):844–852. 10.1097/00004583-199706000-00023 9183141 10.1097/00004583-199706000-00023

[46] Sharma E, Sharma LP, Balachander S, Lin B, Manohar H, Khanna P, Lu C, Garg K, Thomas TL, Au ACL, Selles RR, Højgaard DRMA, Skarphedinsson G, Stewart SE (2021) Comorbidities in obsessive-compulsive disorder across the lifespan: A systematic review and meta-analysis. Front Psychiatry. 10.3389/fpsyt.2021.703701 34858219 10.3389/fpsyt.2021.703701 PMC8631971

[47] Snyder HR, Friedman NP, Hankin BL (2021) Associations between task performance and self-report measures of cognitive control: Shared versus distinct abilities. Assessment 28(4):1080–1096. 10.1177/1073191120965694 33084353 10.1177/1073191120965694 PMC8058111

[48] Snyder HR, Kaiser RH, Warren SL, Heller W (2015) Obsessive-compulsive disorder is associated with broad impairments in executive function: A meta-analysis. Clin Psychol Sci 3(2):301–330. 10.1177/2167702614534210 25755918 10.1177/2167702614534210 PMC4351670

[49] Uhre CF, Ritter M, Jepsen JRM, Uhre VF, Lønfeldt NN, Müller AD, Plessen KJ, Vangkilde S, Blair RJ, Pagsberg AK (2023) Atypical neurocognitive functioning in children and adolescents with obsessive–compulsive disorder (OCD). Eur Child Adolesc Psychiatry. 10.1007/s00787-023-02301-w 37917157 10.1007/s00787-023-02301-w PMC11255040

[50] Uhre CF, Uhre VF, Lønfeldt NN, Pretzmann L, Vangkilde S, Plessen KJ, Gluud C, Jakobsen JC, Pagsberg AK (2020) Systematic review and meta-analysis: Cognitive-behavioral therapy for obsessive-compulsive disorder in children and adolescents. J Am Acad Child Adolesc Psychiatry 59(1):1. 10.1016/j.jaac.2019.08.480 31589909 10.1016/j.jaac.2019.08.480

[51] Voderholzer U, Schwartz C, Freyer T, Zurowski B, Thiel N, Herbst N, Wahl K, Kordon A, Hohagen F, Kuelz AK (2013) Cognitive functioning in medication-free obsessive-compulsive patients treated with cognitive-behavioural therapy. J Obsessive Compuls Relat Disord 2(3):241–248. 10.1016/j.jocrd.2013.03.003

[52] Wechsler D (2008) Technical and Interpretive Manual for the Wechsler Adult Intelligence Scale, 4th edn. NCS Pearson, Inc

[53] Wechsler D (2014) Technical and interpretive manual for the Wechsler Intelligence Scale for Children, 5th edn. NCS Pearson, Inc

[54] World Health Organization (1992) International Classification of Diseases, Tenth Revision (ICD-10). World Health Organization. https://apps.who.int/iris/handle/10665/37958

[55] Zandt F, Prior M, Kyrios M (2009) Similarities and differences between children and adolescents with autism spectrum disorder and those with obsessive compulsive disorder: Executive functioning and repetitive behaviour. Autism 13(1):43–57. 10.1177/1362361308097120 19176576 10.1177/1362361308097120

