# Supplementary material for: Parent- and child-reported executive functioning and response to psychotherapy in pediatric obsessive-compulsive disorder: Results from the TECTO study
Source: Eur Child Adolesc Psychiatry. 2026 Mar 28;35(7):2211–23. doi: 10.1007/s00787-026-03013-7 (PMC13427988; doi:10.1007/s00787-026-03013-7)

Supplementary Material:

**Parent- and child-reported executive functioning and response to psychotherapy in pediatric obsessive-compulsive disorder: Results from the TECTO study**

Melanie Ritter^1,2^, Valdemar Uhre, Sofie Heidenheim Christensen, Nicoline Løcke Jepsen Korsbjerg, Nicole Nadine Lønfeldt, Linea Pretzmann, Christine Lykke Thoustrup, Anna-Rosa Cecilie Mora-Jensen, Kerstin Jessica Plessen, Jens Richard Møllegaard Jepsen, Signe Vangkilde, Camilla Funch Uhre, Anne Katrine Pagsberg, Robert James Blair

^1^ Child and Adolescent Mental Health Center, Copenhagen University Hospital – Bispebjerg and Frederiksberg, Copenhagen, Denmark. Postal address: Gentofte Hospitalsvej 3A, 2900 Hellerup, Denmark

^2^ Department of Clinical Medicine, Faculty of Health and Medical Sciences, University of Copenhagen, Copenhagen, Denmark. Postal address: Blegdamsvej 3B, 33.5, Sektion A, 2200 København N, Denmark

**Corresponding author:**

Melanie Ritter. Email: [melanie.ritter@regionh.dk](mailto:melanie.ritter@regionh.dk)

**Content**

[1 Methods 3](#_Toc208924092)

[1.1 Measures 3](#_Toc208924093)

[1.1.1 Parent- and Self-Reported Executive Function 3](#_Toc208924094)

[1.2 Participants 4](#_Toc208924095)

[2 Data Analysis 6](#_Toc208924096)

[2.1 Demographic and clinical characteristics 6](#_Toc208924097)

[2.2 Checking Assumptions 7](#_Toc208924098)

[2.2.1 Normality (QQ Plots) 7](#_Toc208924099)

[3 Results 9](#_Toc208924100)

[3.1 Demographic and clinical characteristics 9](#_Toc208924101)

[3.2 Executive Function Pre-Treatment and Associations with Symptom Severity 10](#_Toc208924102)

[3.2.1 Follow-up ANOVA tables 10](#_Toc208924103)

[3.2.2 Sensitivity analyses (including Parental Education and IQ) 11](#_Toc208924104)

[3.2.3 Proportion of Participants with Clinically Elevated Domain Score 12](#_Toc208924105)

[3.2.4 Number of clinically elevated BRIEF-2 domains 13](#_Toc208924106)

[3.2.5 Number of clinically elevated BRIEF-2 domains (T score ≥ 60) 17](#_Toc208924107)

[3.2.6 Number of clinically elevated BRIEF-2 domains (T score ≥ 65) 18](#_Toc208924108)

[3.2.7 Number of clinically elevated BRIEF-2 domains (T score ≥ 70) 19](#_Toc208924109)

[3.2.8 Individual predictors from multiple linear regression 20](#_Toc208924110)

[3.3 Executive Function Change during Treatment and Association with Symptom Change 21](#_Toc208924111)

[3.3.1 Repeated Measures ANOVA, Complete Results 21](#_Toc208924112)

[3.3.2 Proportion of Participants with Clinically Elevated Scores Post-treatment 22](#_Toc208924113)

[3.3.3 Follow-up Estimated Marginal Means (Patients) 22](#_Toc208924114)

[3.3.4 Follow-up Estimated Marginal Means (Controls) 23](#_Toc208924115)

[3.4 Pre-Treatment Executive Function as a Moderator 24](#_Toc208924116)

[3.4.1 Multiple linear regression tables 24](#_Toc208924117)

[3.4.2 Plots of predictors and interactions 25](#_Toc208924118)

# Methods

## Measures

### Parent- and Self-Reported Executive Function

**Table S1** Description of BRIEF-II Clinical Scales

| Clinical Scale | Number of Items | | Rated Behavior |
| --- | --- | --- | --- |
|  | Parent-Report | Self-Report |  |
| Inhibit | 8 | 8 | Controlling impulses, stop behavior at the right moment |
| Shift | 8 | 8 | Shifting between different situations/activities or from one part of a problem to another, adapting to circumstances; shifting; flexible problem solving |
| Emotional Control | 8 | 6 | Moderate emotional control in a suiting manner |
| Self-Monitor | 4 | 5 | Monitoring effect of one’s own behavior |
| Initiate | 4 | - | Initiate task/activity; generate new ideas |
| Task-Completion | - | 7 | Finishing schoolwork/domestic duties in time; completing tasks within a time frame; working in a sufficient tempo |
| Working Memory | 8 | 8 | Keeping information during finishing a task; continuing an activity |
| Plan/Organize | 8 | 10 | Foresee incidents; set goals; planning relevant steps ahead of fulfilling a task; systematical execution of tasks; understanding/communicating a central idea/concept |
| Task-Monitor | 6 | - | Controlling ones work; evaluating work during/after a task to assure fulfilling the purpose of the task |
| Organization of Materials | 5 | - | Organizing working space, playing area, and materials in a good way |

*Note*: Source: BRIEF-2 Manual (Gioia et al., 2015)

## Participants

For parent-ratings (covering age 8-17 years), 112 patients and 74 controls were included, and of these, 69 patients and 67 controls also participated with follow-up data. For self-ratings (covering age 11-17 years), 82 patients and 52 controls were included, and of these, 49 patients and 49 controls additionally participated with follow-up data.

**Figure S1** Participant Flow (Parent-report)


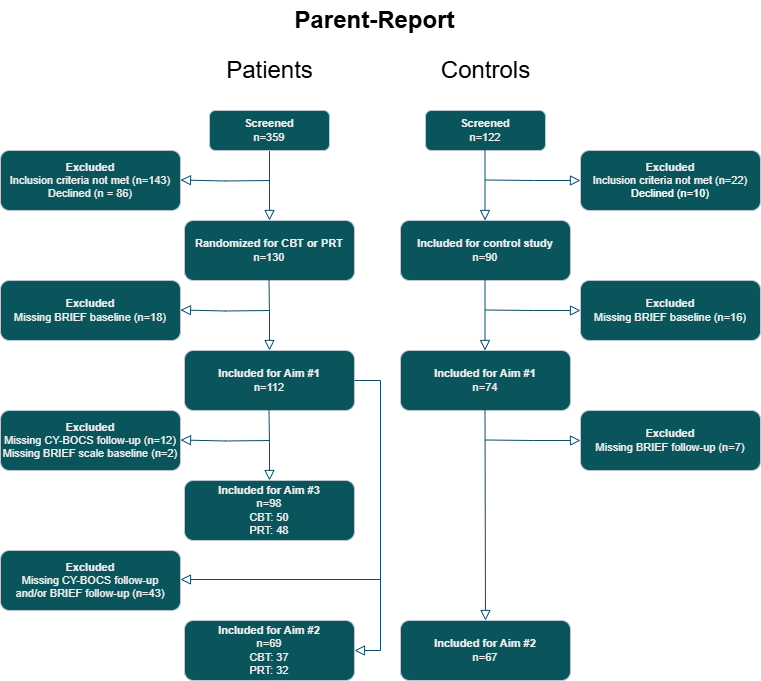


**Figure S2** Participant Flow (Self-report)


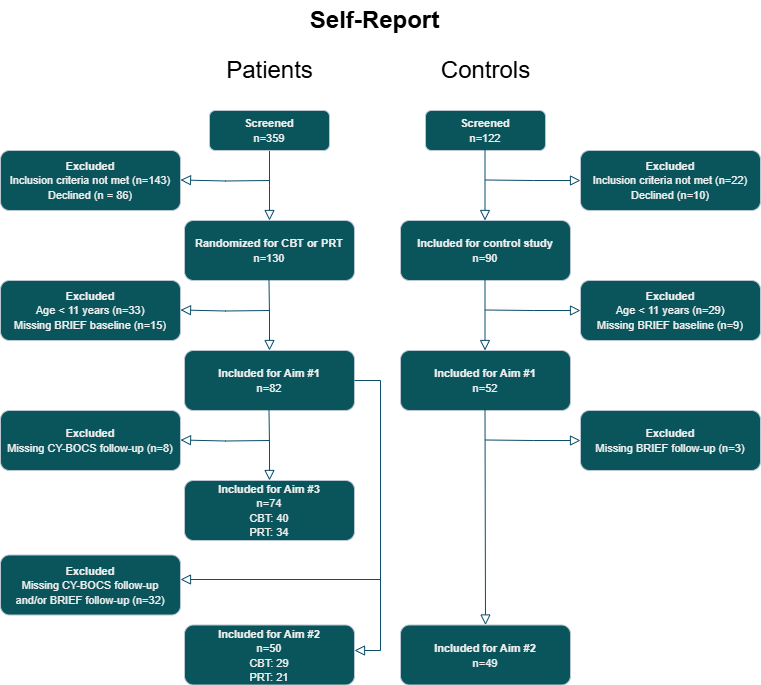


# Data Analysis

Statistical analyses were performed with the statistical software R (version 4.5.0). We conducted complete case analyses for each statistical test (for details about amount of missing data, see Figure S1 and S2). Treatment allocation was represented by a binary variable (PRT = 0, CBT = 1. Analyses were conducted separately for parent-rated (N=112 patients and 74 controls) and self-rated data (N=82 patients and 52 controls). For cases with two parent ratings available, parent-mean T-scores were used in analyses – interrater-correlation between parents has been reported as moderate to high in both normative and clinical samples (Gioia et al., 2018), and for the current sample (patients and controls at baseline), parent agreement was high across domains (*r* = 0.63-0.77, all *p* < .001). An alpha level of .05 was used for all statistical tests. Multiple linear regression models were fitted using the base lm() function in R. All BRIEF-2 domains were inspected for normality via QQ-plots (See Supplementary Figures S3-S6).

To correct for multiple comparisons, the Benjamini-Hochberg (BH) procedure with a cut-off for false discovery rate (FDR) of 5 % was used within each main analysis. Critical values were 0.01281 for parent-ratings and 0.00567 for self-ratings. Since parent- and child reports often differ in clinical studies, we applied the BH procedure for the two informants separately.

For the main analyses, we test five hypotheses (aims 1.1, 1.2, 2.1, 2.2, and 3), and thus the BH correction is based on the five corresponding p values. For main analyses of parent-report data, we get the following p values:

$2.2\times e^{-16}$, 0.04396, $2.375\times e^{-06}$, 0.467, 0.007686

and a critical value of 0.01281.

For main analyses of self-report data, we get the following p values:

$3.009\times e^{-08}$, 0.001131, $5.252\times e^{-05}$, 0.631, 0.004536

and a critical value of 0.00567.

## Demographic and clinical characteristics

Potential differences in baseline characteristics of patients versus controls were tested using independent-samples *t*-tests for continuous variables and chi-square tests for binary variables. Following previous work (Hybel et al., 2017; Uhre et al., 2023), years of parental education was calculated as either the mean of both parents, or, if only one parent participated, that parent’s value was used. We reported the comorbid psychiatric diagnoses that were most frequently present in our sample (> 3 %).

## Checking Assumptions

### Normality (QQ Plots)

**Supplementary Figure S3** QQ Plot of BRIEF sub-scales for Patients (Parent-Report; child age: 8-17)


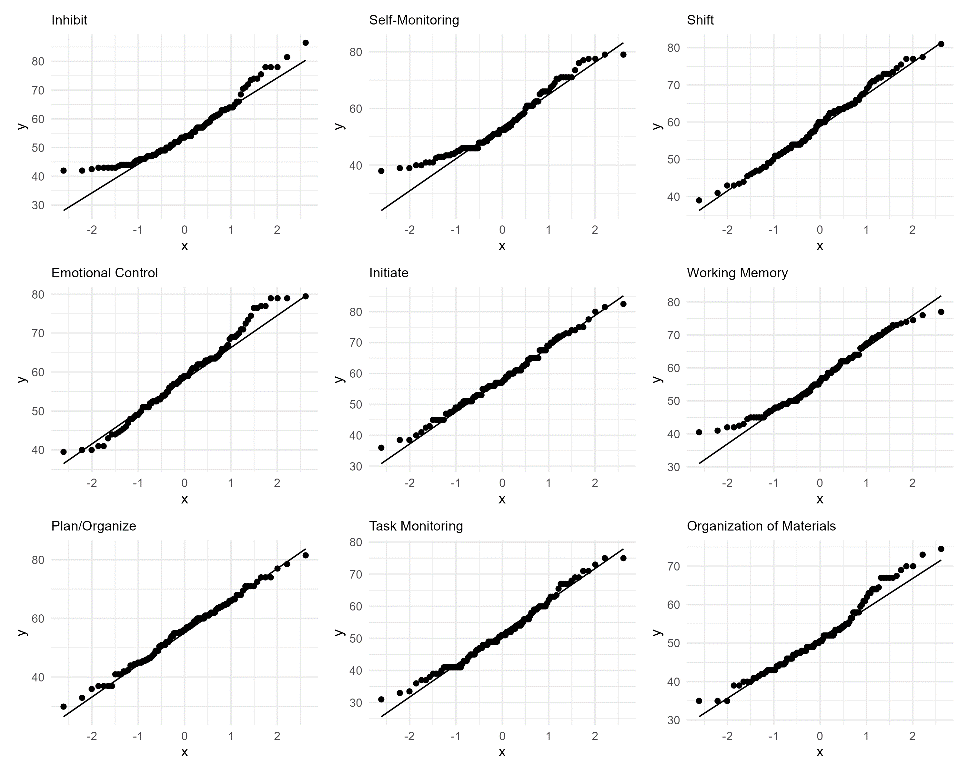


**Supplementary Figure S4** QQ Plot of BRIEF sub-scales for Controls (Parent-Report; child age: 8-17)


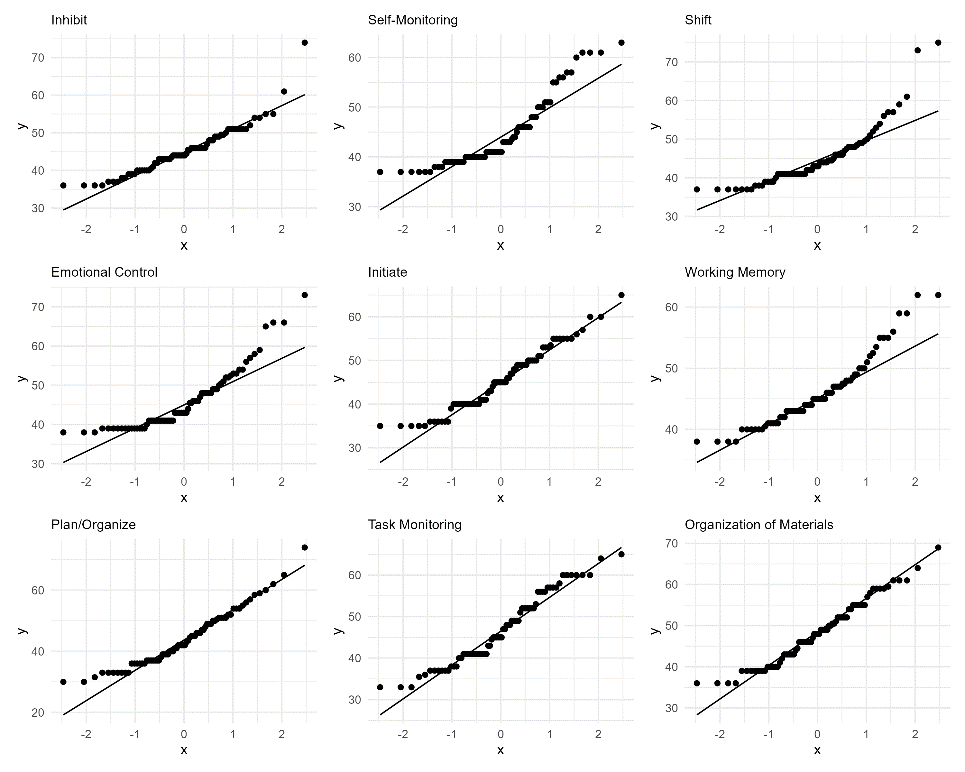


**Supplementary Figure S5** QQ Plot of BRIEF sub-scales for Patients (Self-Report; child age: 11-17)


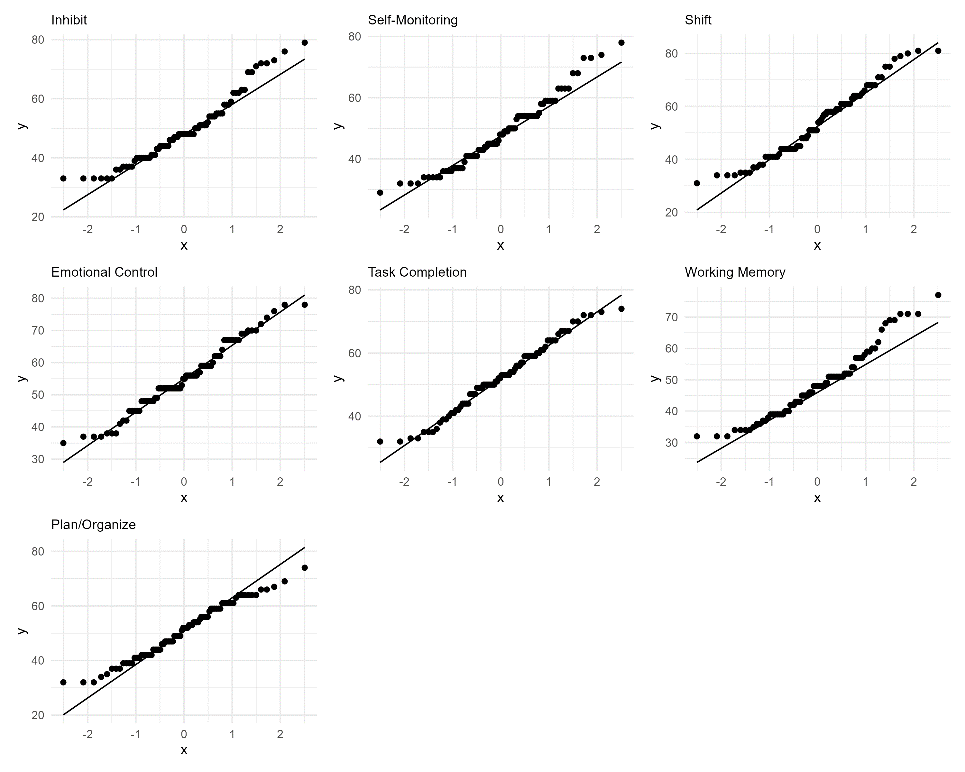


**Supplementary Figure S6** QQ Plot of BRIEF sub-scales for Controls (Self-Report; child age: 11-17)


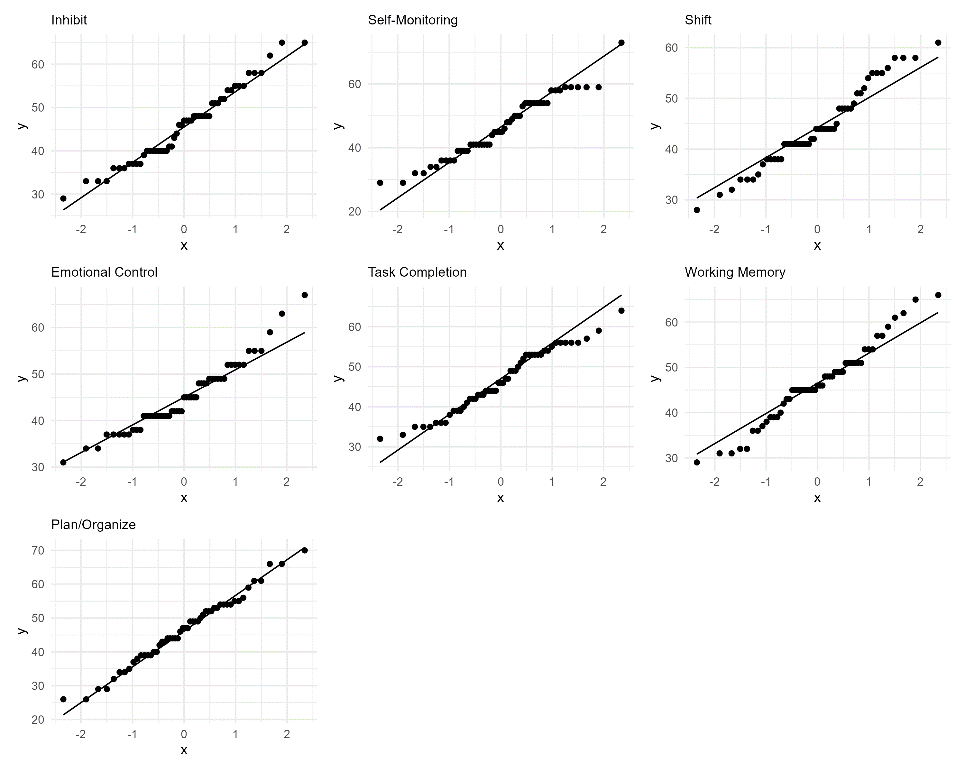


# Results

## Demographic and clinical characteristics

**Supplementary Table S2** Demographic and Clinical Characteristics pre-treatment for patients with complete BRIEF data at pre- and post-treatment (Parent-Report and Self-Report)

|  |  | **OCD**  Mean(*SD*), *n*(%) |  | | **CBT**  Mean(*SD*), *n*(%) | **PRT**  Mean(*SD*), *n*(%) | **CBT vs PRT**  *p*; Effect | |
| --- | --- | --- | --- | --- | --- | --- | --- | --- |
| Number of participants  Parent-report  Self-report |  | 69  50 |  |  | 37  29 | 32  21 |  |  |
| Number of females  Parent-report  Self-report |  | 41 (59.4)  34 (68.0) |  |  | 23 (62.2)  10 (34.5) | 18 (56.3)  15 (71.4) | .800  .893 | .060  .063 |
| Age in years  Parent-report  Self-report |  | 13.1 (2.74)  14.5 (1.94) |  |  | 12.9 (2.77)  14.3 (2.00) | 13.3 (2.73)  14.8 (1.86) | .631  .386 | .117  .248 |
| Parental education, years  Parent-report  Self-report |  | 15.6 (2.20)  15.4 (2.22) |  |  | 15.3 (2.56)  15.3 (2.49) | 15.9 (1.60)  15.6 (1.75) | .292  .682 | .250  .114 |
| Intelligence Quotient (IQ)  Parent-report  Self-report |  | 102 (12.9)  101 (11.4) |  |  | 101 (12.8)  101 (11.4) | 103 (13.2)  101 (11.7) | .565  .804 | .140  .072 |
| CY-BOCS Pre-Treatment Total Score  Parent-report  Self-report |  | 24.5 (3.92)  24.6 (4.00) |  |  | 24.1 (3.93)  24.1 (3.78) | 24.9 (3.94)  25.1 (4.30) | .439  .804 | .188  .251 |
| Co-morbid disorders |  |  |  |  |  |  |  |  |
| Asperger’s (F84.5)  Parent-report  Self-report |  | 9 (13.0)  6 (12.77) |  |  | 3 (8.1)  2 (7.7) | 6 (18.7)  4 (19.0) | -  - | -  - |
| ADHD (F90.0)  Parent-report  Self-report |  | 9 (13.0)  4 (8.51) |  |  | 5 (13.5)  3 (11.5) | 4 (12.5)  1 (4.8) | -  - | -  - |
| GAD (F41.1 & F93.8)  Parent-report  Self-report |  | 5 (7.25)  3 (6.38) |  |  | 1 (2.7)  0 (0) | 4 (12.5)  3 (14.3) | -  - | -  - |
| Tourette’s (F95.2)  Parent-report  Self-report |  | 4 (5.80)  3 (6.38) |  |  | 2 (5.4)  1 (3.8) | 2 (6.25)  2 (9.5) | -  - | -  - |
| Any co-morbid disorder  Parent-report  Self-report |  | 38 (55.07)  25 (50.0) |  |  | 20 (54.05)  14 (48.3) | 18 (56.25)  11 (52.4) | -  - | -  - |

*Note*: OCD = Patients with OCD. CTR = Control children. CBT = Patients who received cognitive-behavioral therapy (CBT). PRT = Patients who received psychoeducation and relaxation training (PRT). Effect: As effect sizes we used Cohens *d* for numerical data and Cramer’s *V* for categorical data. Comorbid diagnosis is reported if present in > 3 % of patients (self-report/parent-report). ADHD = Attention-Deficit/Hyperactivity Disorder. GAD = Generalized Anxiety Disorder.

## Executive Function Pre-Treatment and Associations with Symptom Severity

### Follow-up ANOVA tables

**Supplementary Table S3** Follow-Up ANOVAs of BRIEF domains pre-treatment (OCD vs. controls; parent-report; child age: 8-17)

|  | **OCD**  Estimated Mean | **CTR**  Estimated Mean | F(9,174) | *p* | Cohen’s *d* |
| --- | --- | --- | --- | --- | --- |
| BRIEF-II sub-domain |  |  |  |  |  |
| Inhibition | 55.28 | 45.25 | 71.30 | **<.001** | 1.16 |
| Self-Monitoring | 54.66 | 44.60 | 61.85 | **<.001** | 1.09 |
| Flexibility | 59.31 | 45.04 | 139.38 | **<.001** | 1.70 |
| Emotional Control | 58.81 | 45.97 | 104.28 | **<.001** | 1.46 |
| Initiating | 58.46 | 45.53 | 108.59 | **<.001** | 1.46 |
| Working Memory | 56.78 | 45.95 | 101.90 | **<.001** | 1.38 |
| Planning/Organizing | 55.84 | 44.10 | 65.24 | **<.001** | 1.18 |
| Task Monitoring | 51.71 | 46.89 | 12.97 | **<.001** | 0.52 |
| Organization of Materials | 51.79 | 48.36 | 8.19 | **.005** | 0.42 |

*Note*: OCD = Patients with OCD. CTR = Non-psychiatric control children.

**Supplementary Table S4** Follow-Up ANOVAs of BRIEF domains pre-treatment (OCD vs. controls; self-report; child age: 11-17)

|  | **OCD**  Estimated Mean | **CTR**  Estimated Mean | F (7,126) | *p* | Cohen’s *d* |
| --- | --- | --- | --- | --- | --- |
| BRIEF-II sub-domain |  |  |  |  |  |
| Inhibition | 49.22 | 45.74 | 4.30 | **.040** | 0.35 |
| Self-Monitoring | 48.35 | 46.35 | 1.24 | .267 | 0.19 |
| Flexibility | 53.55 | 44.21 | 27.84 | **<.001** | 0.84 |
| Emotional Control | 55.25 | 45.06 | 45.23 | **<.001** | 1.11 |
| Task Completion | 52.32 | 46.45 | 13.90 | **<.001** | 0.62 |
| Working Memory | 48.30 | 46.38 | 1.39 | .241 | 0.20 |
| Planning/Organizing | 51.00 | 46.44 | 6.55 | **.012** | 0.46 |

*Note*: OCD = Patients with OCD. CTR = Non-psychiatric control children.

### Sensitivity analyses (including Parental Education and IQ)

The MANCOVA analyses with IQ and parental education as covariates continued to indicate a significant multivariate effect of group (OCD versus controls) on the combined BRIEF clinical scales for both self-report and parent-report (Pillai’s Trace = 0.350, F(7,75) = 5.781, p <.001, ${\eta_{p}}^{2}$ = 0.33; Pillai’s Trace = 0.543, F(9,109) = 14.372, p <.001, ${\eta_{p}}^{2}$ = 0.52). The multiple linear regression remained significant for the self-report (F(9,65) = 3.662, *p* <.001, R^2^ = .336) and insignificant for the parent-report data (F(11,89) = 1.789, *p* = .068, R^2^ = .181).

**Supplementary Table S5** Follow-Up ANOVA’s with IQ and Parental Education (OCD vs. controls; parent-report; child age: 8-17)

| BRIEF-II sub-domain | *F*(9,109) | *p* |
| --- | --- | --- |
|  |  |  |
| Inhibition | 57.559 | **<.001** |
| Self-Monitoring | 24.793 | **<.001** |
| Flexibility | 71.697 | **<.001** |
| Emotional Control | 54.412 | **<.001** |
| Initiating | 52.338 | **<.001** |
| Working Memory | 66.267 | **<.001** |
| Planning/Organizing | 32.726 | **<.001** |
| Task Monitoring | 13.500 | **<.001** |
| Organization of Materials | 6.126 | **.015** |

**Supplementary Table S6** Follow-Up ANOVA’s with IQ and Parental Education (OCD vs. controls; self-report; child age: 11-17)

| BRIEF-II sub-domain | *F*(7,75) | *p* |
| --- | --- | --- |
|  |  |  |
| Inhibition | 4.930 | **.021** |
| Self-Monitoring | 1.931 | .169 |
| Flexibility | 10.220 | **.002** |
| Emotional Control | 35.544 | **<.001** |
| Task Completion | 10.900 | **.001** |
| Working Memory | 1.015 | .317 |
| Planning/Organizing | 2.630 | .109 |

**Supplementary Table S7** Multiple Regression with IQ and Parental Education predicting CY-BOCS baseline (self-report; child age: 11-17)

| Independent variable | Regression coefficient | *t* | *p* |
| --- | --- | --- | --- |
| Intercept | 12.660 | 2.135 | .037 |
| BRIEF-II sub-domain |  |  |  |
| Inhibition | -0.097 | -1.480 | .144 |
| Self-Monitoring | -0.143 | -2.450 | .017 |
| Flexibility | 0.233 | 4.457 | **<.001** |
| Emotional Control | 0.028 | 0.446 | .673 |
| Task Completion | -0.013 | -0.182 | .856 |
| Working Memory | 0.011 | 0.143 | .887 |
| Planning/Organizing | 0.053 | 0.665 | .211 |

### Proportion of Participants with Clinically Elevated Domain Score

**Supplementary Table S8** Proportion of Participants with Clinically Elevated Scores (Parent-Rating)

| **Scale** | **0-59** | **60-64** | **65-69** | **70+** |
| --- | --- | --- | --- | --- |
| Inhibit | 79 (71.2%) | 16 (14.4%) | 4 (3.6%) | 12 (10.8%) |
| Self-Monitor | 77 (68.8%) | 11 (9.8%) | 10 (8.9%) | 14 (12.5%) |
| Shift | 54 (50%) | 27 (25%) | 11 (10.2%) | 16 (14.8%) |
| Emotional Control | 61 (55.5%) | 25 (22.7%) | 10 (9.1%) | 14 (12.7%) |
| Initiate | 62 (56.4%) | 17 (15.5%) | 14 (12.7%) | 17 (15.5%) |
| Working Memory | 71 (64%) | 19 (17.1%) | 9 (8.1%) | 12 (10.8%) |
| Planning/Organizing | 67 (62%) | 21 (19.4%) | 9 (8.3%) | 11 (10.2%) |
| Task-Monitor | 87 (78.4%) | 11 (9.9%) | 8 (7.2%) | 5 (4.5%) |
| Organization of Materials | 91 (82%) | 9 (8.1%) | 7 (6.3%) | 4 (3.6%) |

**Supplementary Table S9** Proportion of Participants with Clinically Elevated Scores (Self-Rating)

| **Scale** | **0-59** | **60-64** | **65-69** | **70+** |
| --- | --- | --- | --- | --- |
| Inhibit | 69 (84.1%) | 5 (6.1%) | 2 (2.4%) | 6 (7.3%) |
| Self-Monitor | 72 (87.8%) | 4 (4.9%) | 2 (2.4%) | 4 (4.9%) |
| Shift | 56 (68.3%) | 11 (13.4%) | 6 (7.3%) | 9 (11%) |
| Emotional Control | 59 (72%) | 6 (7.3%) | 9 (11%) | 8 (9.8%) |
| Task-Completion | 63 (76.8%) | 9 (11%) | 4 (4.9%) | 6 (7.3%) |
| Working Memory | 71 (86.6%) | 3 (3.7%) | 4 (4.9%) | 4 (4.9%) |
| Planning/Organizing | 64 (78%) | 13 (15.9%) | 4 (4.9%) | 1 (1.2%) |

### Number of clinically elevated BRIEF-2 domains

**Table S10** Number of clinically elevated BRIEF-2 domains

| **ClinGr** | **n_elevated** | **n** | **Percent** | **Cutoff** | **Report** |
| --- | --- | --- | --- | --- | --- |
| 1 | 0 | 19 | 17.3 | ≥60 | Parent |
| 1 | 1 | 19 | 17.3 | ≥60 | Parent |
| 1 | 2 | 16 | 14.5 | ≥60 | Parent |
| 1 | 3 | 14 | 12.7 | ≥60 | Parent |
| 1 | 4 | 10 | 9.1 | ≥60 | Parent |
| 1 | 5 | 8 | 7.3 | ≥60 | Parent |
| 1 | 6 | 7 | 6.4 | ≥60 | Parent |
| 1 | 7 | 5 | 4.5 | ≥60 | Parent |
| 1 | 8 | 8 | 7.3 | ≥60 | Parent |
| 1 | 9 | 4 | 3.6 | ≥60 | Parent |
| 2 | 0 | 56 | 75.7 | ≥60 | Parent |
| 2 | 1 | 12 | 16.2 | ≥60 | Parent |
| 2 | 2 | 1 | 1.4 | ≥60 | Parent |
| 2 | 3 | 2 | 2.7 | ≥60 | Parent |
| 2 | 4 | 2 | 2.7 | ≥60 | Parent |
| 2 | 8 | 1 | 1.4 | ≥60 | Parent |
| 2 | 5 | 0 | 0.0 | ≥60 | Parent |
| 2 | 6 | 0 | 0.0 | ≥60 | Parent |
| 2 | 7 | 0 | 0.0 | ≥60 | Parent |
| 2 | 9 | 0 | 0.0 | ≥60 | Parent |
| 1 | 0 | 52 | 47.3 | ≥65 | Parent |
| 1 | 1 | 18 | 16.4 | ≥65 | Parent |
| 1 | 2 | 8 | 7.3 | ≥65 | Parent |
| 1 | 3 | 9 | 8.2 | ≥65 | Parent |
| 1 | 4 | 6 | 5.5 | ≥65 | Parent |
| 1 | 5 | 7 | 6.4 | ≥65 | Parent |
| 1 | 6 | 5 | 4.5 | ≥65 | Parent |
| 1 | 7 | 2 | 1.8 | ≥65 | Parent |
| 1 | 8 | 3 | 2.7 | ≥65 | Parent |
| 2 | 0 | 68 | 91.9 | ≥65 | Parent |
| 2 | 1 | 3 | 4.1 | ≥65 | Parent |
| 2 | 2 | 1 | 1.4 | ≥65 | Parent |
| 2 | 3 | 1 | 1.4 | ≥65 | Parent |
| 2 | 4 | 1 | 1.4 | ≥65 | Parent |
| 2 | 5 | 0 | 0.0 | ≥65 | Parent |
| 2 | 6 | 0 | 0.0 | ≥65 | Parent |
| 2 | 7 | 0 | 0.0 | ≥65 | Parent |
| 2 | 8 | 0 | 0.0 | ≥65 | Parent |
| 1 | 9 | 0 | 0.0 | ≥65 | Parent |
| 2 | 9 | 0 | 0.0 | ≥65 | Parent |
| 1 | 0 | 66 | 60.0 | ≥70 | Parent |
| 1 | 1 | 19 | 17.3 | ≥70 | Parent |
| 1 | 2 | 10 | 9.1 | ≥70 | Parent |
| 1 | 3 | 6 | 5.5 | ≥70 | Parent |
| 1 | 4 | 4 | 3.6 | ≥70 | Parent |
| 1 | 5 | 1 | 0.9 | ≥70 | Parent |
| 1 | 6 | 2 | 1.8 | ≥70 | Parent |
| 1 | 7 | 1 | 0.9 | ≥70 | Parent |
| 1 | 8 | 1 | 0.9 | ≥70 | Parent |
| 2 | 0 | 70 | 94.6 | ≥70 | Parent |
| 2 | 1 | 3 | 4.1 | ≥70 | Parent |
| 2 | 2 | 1 | 1.4 | ≥70 | Parent |
| 2 | 3 | 0 | 0.0 | ≥70 | Parent |
| 2 | 4 | 0 | 0.0 | ≥70 | Parent |
| 2 | 5 | 0 | 0.0 | ≥70 | Parent |
| 2 | 6 | 0 | 0.0 | ≥70 | Parent |
| 2 | 7 | 0 | 0.0 | ≥70 | Parent |
| 2 | 8 | 0 | 0.0 | ≥70 | Parent |
| 1 | 9 | 0 | 0.0 | ≥70 | Parent |
| 2 | 9 | 0 | 0.0 | ≥70 | Parent |
| 1 | 0 | 38 | 46.3 | ≥60 | Child |
| 1 | 1 | 16 | 19.5 | ≥60 | Child |
| 1 | 2 | 7 | 8.5 | ≥60 | Child |
| 1 | 3 | 9 | 11.0 | ≥60 | Child |
| 1 | 4 | 3 | 3.7 | ≥60 | Child |
| 1 | 5 | 4 | 4.9 | ≥60 | Child |
| 1 | 6 | 4 | 4.9 | ≥60 | Child |
| 1 | 7 | 1 | 1.2 | ≥60 | Child |
| 2 | 0 | 41 | 78.8 | ≥60 | Child |
| 2 | 1 | 8 | 15.4 | ≥60 | Child |
| 2 | 2 | 1 | 1.9 | ≥60 | Child |
| 2 | 3 | 1 | 1.9 | ≥60 | Child |
| 2 | 4 | 1 | 1.9 | ≥60 | Child |
| 2 | 5 | 0 | 0.0 | ≥60 | Child |
| 2 | 6 | 0 | 0.0 | ≥60 | Child |
| 2 | 7 | 0 | 0.0 | ≥60 | Child |
| 1 | 0 | 50 | 61.0 | ≥65 | Child |
| 1 | 1 | 14 | 17.1 | ≥65 | Child |
| 1 | 2 | 8 | 9.8 | ≥65 | Child |
| 1 | 3 | 5 | 6.1 | ≥65 | Child |
| 1 | 4 | 2 | 2.4 | ≥65 | Child |
| 1 | 5 | 2 | 2.4 | ≥65 | Child |
| 1 | 6 | 1 | 1.2 | ≥65 | Child |
| 2 | 0 | 44 | 84.6 | ≥65 | Child |
| 2 | 1 | 7 | 13.5 | ≥65 | Child |
| 2 | 2 | 1 | 1.9 | ≥65 | Child |
| 2 | 3 | 0 | 0.0 | ≥65 | Child |
| 2 | 4 | 0 | 0.0 | ≥65 | Child |
| 2 | 5 | 0 | 0.0 | ≥65 | Child |
| 2 | 6 | 0 | 0.0 | ≥65 | Child |
| 1 | 7 | 0 | 0.0 | ≥65 | Child |
| 2 | 7 | 0 | 0.0 | ≥65 | Child |
| 1 | 0 | 62 | 75.6 | ≥70 | Child |
| 1 | 1 | 9 | 11.0 | ≥70 | Child |
| 1 | 2 | 6 | 7.3 | ≥70 | Child |
| 1 | 3 | 3 | 3.7 | ≥70 | Child |
| 1 | 4 | 2 | 2.4 | ≥70 | Child |
| 2 | 0 | 50 | 96.2 | ≥70 | Child |
| 2 | 1 | 2 | 3.8 | ≥70 | Child |
| 2 | 2 | 0 | 0.0 | ≥70 | Child |
| 2 | 3 | 0 | 0.0 | ≥70 | Child |
| 2 | 4 | 0 | 0.0 | ≥70 | Child |
| 1 | 5 | 0 | 0.0 | ≥70 | Child |
| 2 | 5 | 0 | 0.0 | ≥70 | Child |
| 1 | 6 | 0 | 0.0 | ≥70 | Child |
| 2 | 6 | 0 | 0.0 | ≥70 | Child |
| 1 | 7 | 0 | 0.0 | ≥70 | Child |
| 2 | 7 | 0 | 0.0 | ≥70 | Child |

### Number of clinically elevated BRIEF-2 domains (T score ≥ 60)

**Figure S7** Number of clinically elevated BRIEF-2 domains (T score ≥ 60)


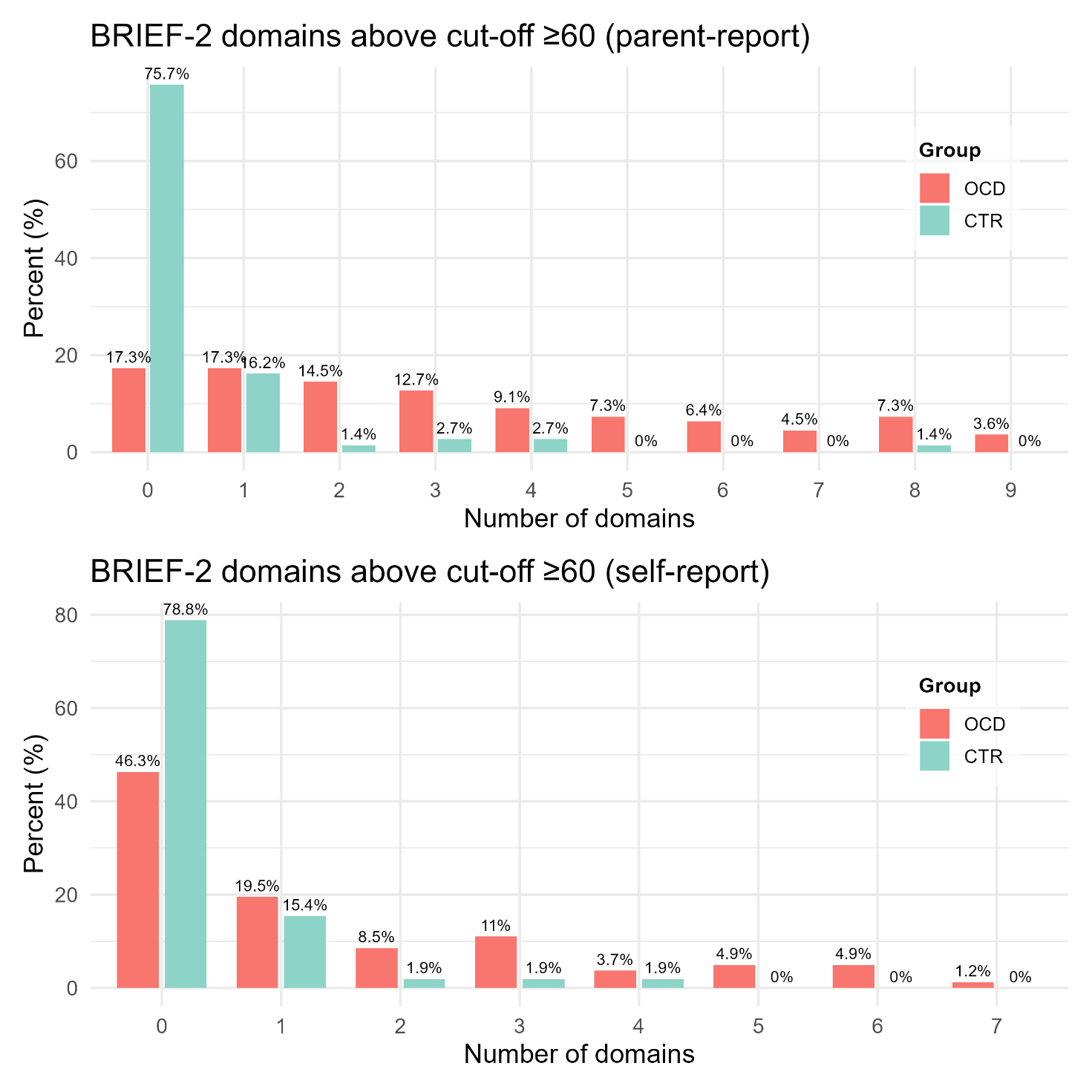


*Note*: OCD = Patients with OCD. CTR = Control children.

### Number of clinically elevated BRIEF-2 domains (T score ≥ 65)

**Figure S8** Number of clinically elevated BRIEF-2 domains (T score ≥ 65)


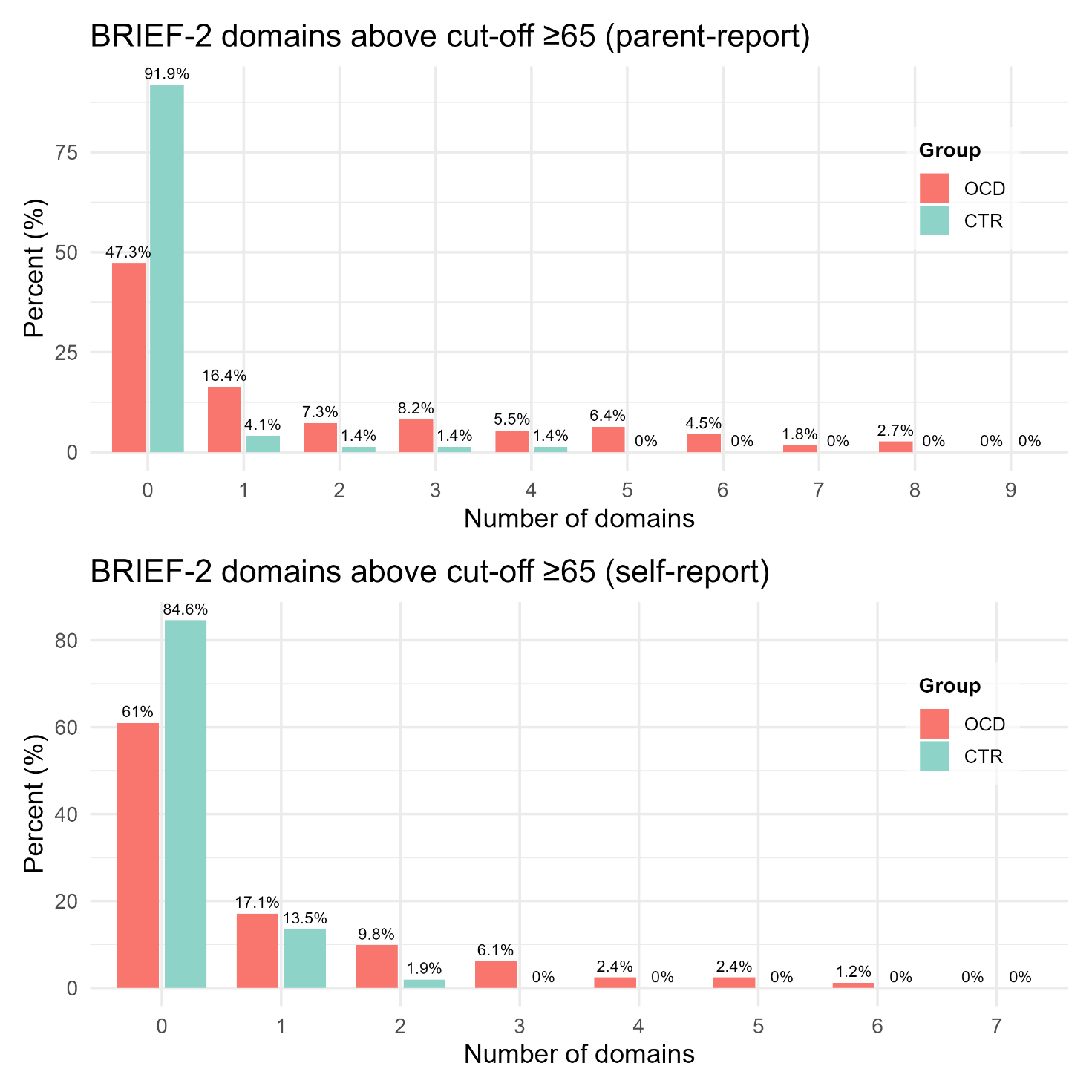


*Note*: OCD = Patients with OCD. CTR = Control children.

### Number of clinically elevated BRIEF-2 domains (T score ≥ 70)

**Figure S9** Number of clinically elevated BRIEF-2 domains (T score ≥ 70)


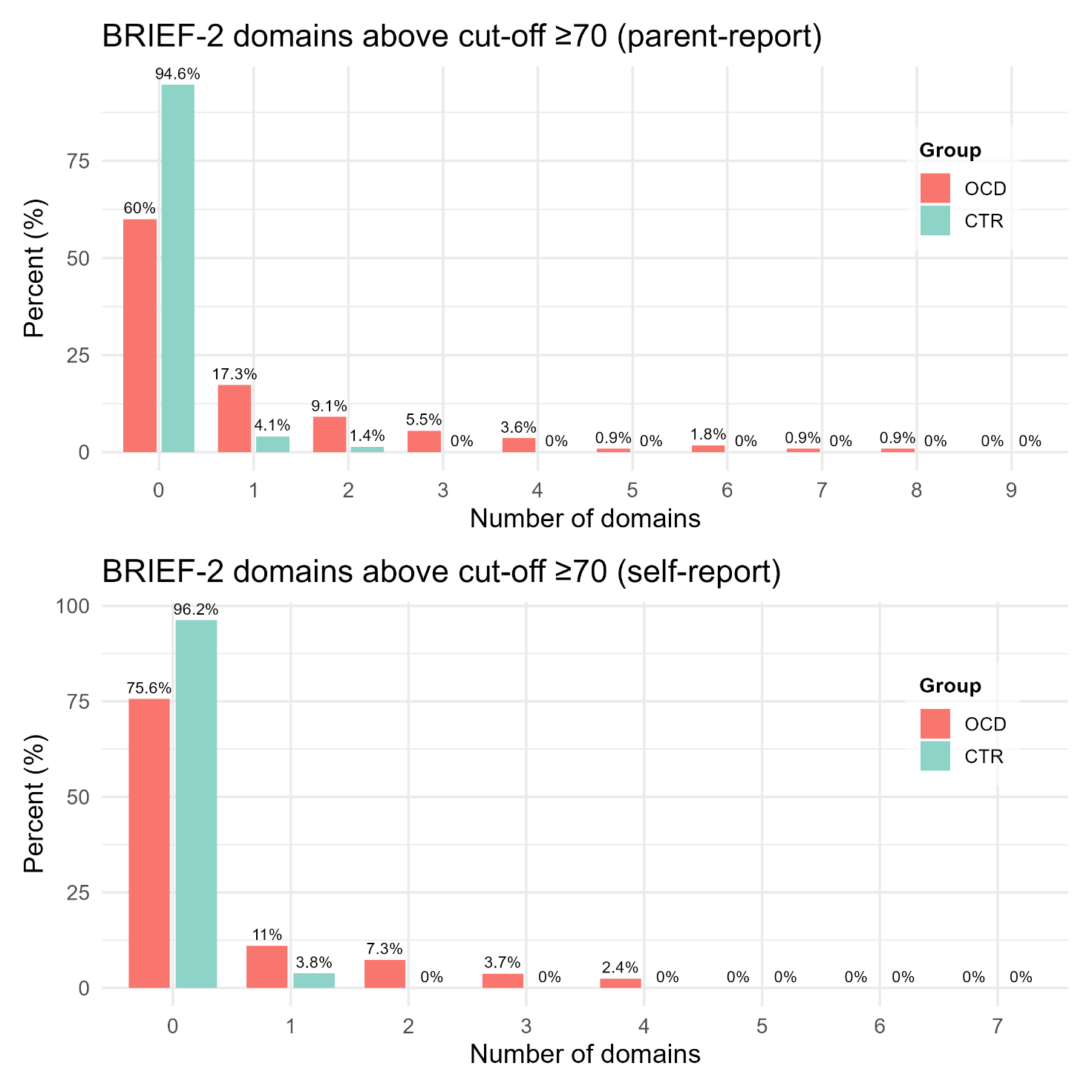


*Note*: OCD = Patients with OCD. CTR = Control children.

### Individual predictors from multiple linear regression

**Supplementary Table S11** Multiple Linear Regression Results (self-report; child age: 11-17)

| Independent variable | Regression coefficient | *t* | *p* |
| --- | --- | --- | --- |
| Intercept | 20.975 | 6.971 | **<.001** |
| BRIEF-II sub-domain |  |  |  |
| Inhibition | -0.115 | -1.850 | .068 |
| Self-Monitoring | -0.114 | -2.018 | .047 |
| Flexibility | 0.207 | 4.272 | **<.001** |
| Emotional Control | 0.037 | 0.648 | .518 |
| Task Completion | -0.032 | -0.511 | .611 |
| Working Memory | 0.046 | 0.638 | .526 |
| Planning/Organizing | 0.034 | 0.441 | .661 |

## Executive Function Change during Treatment and Association with Symptom Change

### Repeated Measures ANOVA, Complete Results

**Table S12** 2×2×9 Repeated Measures ANOVA (parent-report; child age: 8-17)

| **Effect** | ***df*** | ***F*** | ***p*** | $\boldsymbol{\eta}_{\boldsymbol{p}}^{\boldsymbol{2}}$ |
| --- | --- | --- | --- | --- |
| Treatment Group | 1.00, 67.00 | 0.891 | .766 | 0.00 |
| Time | 1.00, 67.00 | 25.336 | **<.001** | 0.27 |
| Treatment Group × Time | 1.00, 67.00 | 0.472 | .495 | 0.01 |
| BRIEF Domain | 5.00, 334.79 | 8.313 | **<.001** | 0.11 |
| Treatment Group × Brief Domain | 5.00, 334.79 | 0.805 | .546 | 0.01 |
| Time × BRIEF Domain | 6.57, 439.95 | 2.596 | **.014** | 0.04 |
| Treatment Group × Time × BRIEF Domain | 6.57, 439.95 | 2.452 | **.020** | 0.04 |

**Table S13** 2×2×7 Repeated Measures ANOVA (parent-report; child age: 8-17)

| **Effect** | ***df*** | ***F*** | ***p*** | $\boldsymbol{\eta}_{\boldsymbol{p}}^{\boldsymbol{2}}$ |
| --- | --- | --- | --- | --- |
| Treatment Group | 1, 43 | 0.065 | .800 | 0.00 |
| Time | 1, 43 | 21.762 | **<.001** | 0.34 |
| Treatment Group × Time | 1, 43 | 1.203 | .279 | 0.03 |
| BRIEF Domain | 4.56, 195.96 | 7.391 | **<.001** | 0.15 |
| Treatment Group × Brief Domain | 4.56, 195.96 | 0.152 | .937 | 0.00 |
| Time × BRIEF Domain | 5.16, 221.75 | 1.089 | .368 | 0.02 |
| Treatment Group × Time × BRIEF Domain | 5.16, 221.75 | 1.696 | .134 | 0.04 |

**Figure S10** Mean change in BRIEF Domain T-scores by Treatment Group (Heat Plot)


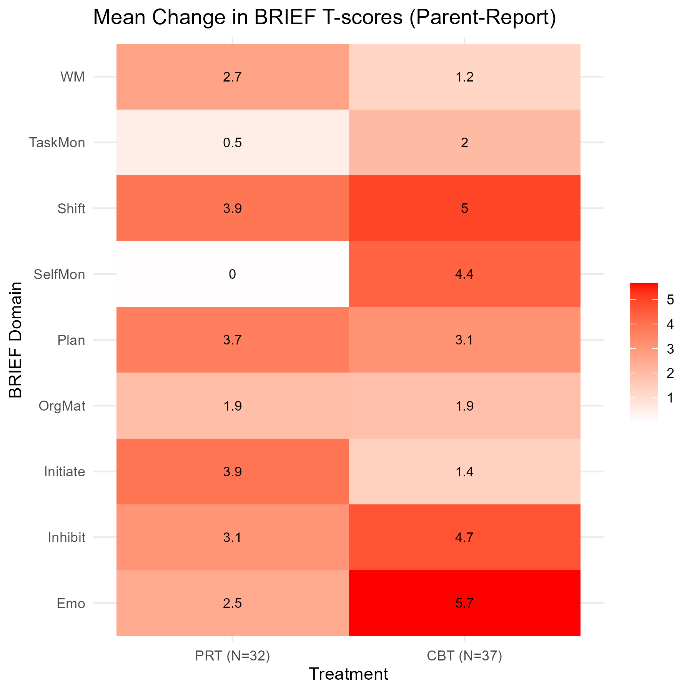

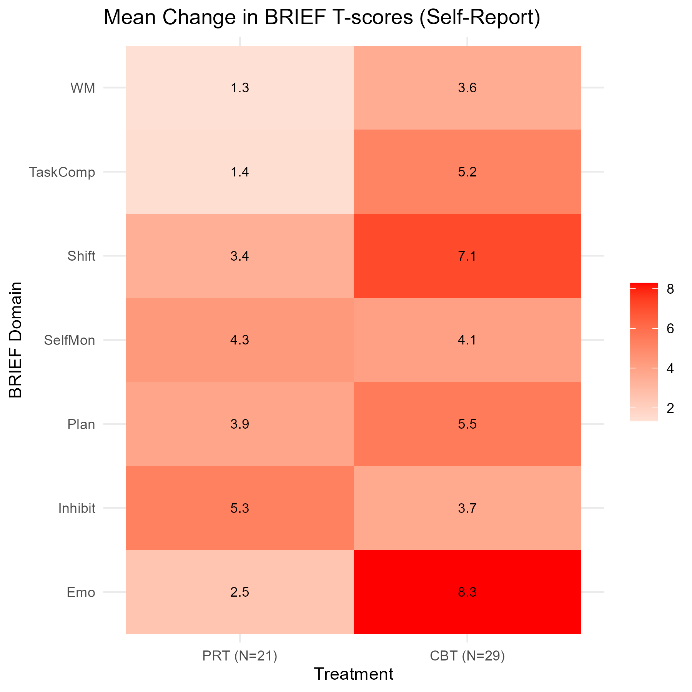


### Proportion of Participants with Clinically Elevated Scores Post-treatment

**Figure S11** Clinically Elevated BRIEF Scores (Patients, post-treatment)


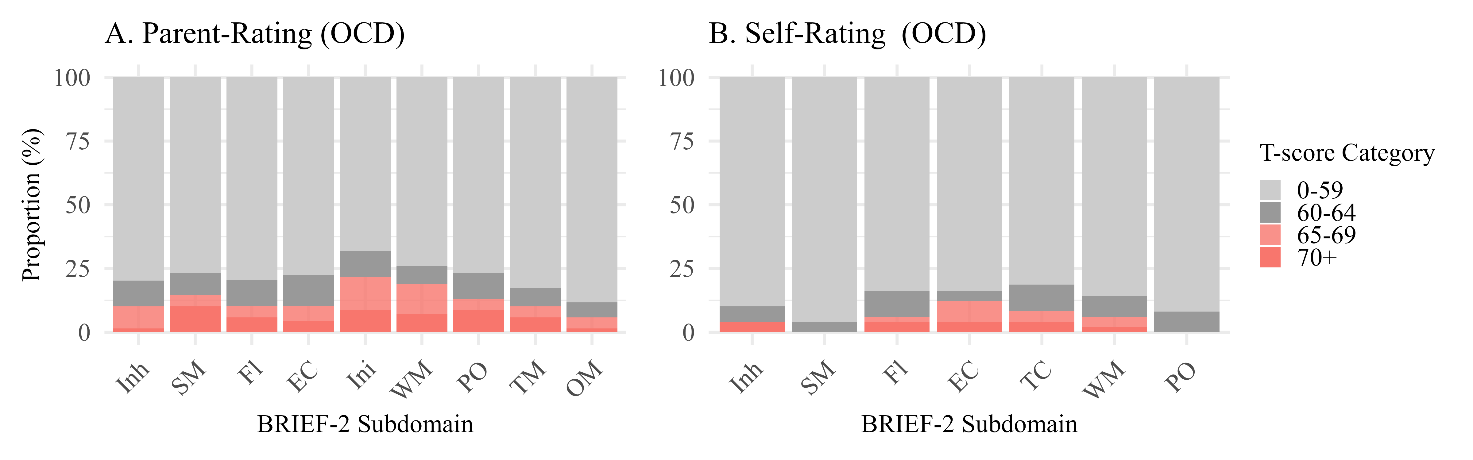


*Note*: BRIEF-2 = Behavior Rating Inventory of Executive Function, Second Edition (Gioia et al., 2015). Inh = Inhibition, SM = Self-Monitoring, Fl = Flexibility, EC = Emotional Control, Ini = Initiating, WM = Working Memory, PO = Planning/Organizing, TC = Task Completion.

### Follow-up Estimated Marginal Means (Patients)

The *emmeans* package in R was used (Lenth et al., 2025).

**Table S14** 2×2×9 Follow-up Estimated Marginal Means (parent-report; child age: 8-17)

|  | **PRT**  Pre-treatment  M(SE) [95 % CI] | **PRT**  Post-treatment  M(SE) [95 % CI] | **CBT**  Pre-treatment  M(SE) [95 % CI] | **CBT**  Post-treatment  M(SE) [95 % CI] |
| --- | --- | --- | --- | --- |
| BRIEF-II Sub Domain |  |  |  |  |
| Inhibition | 53.5 (1.6) [50.4, 56.6] | 50.4 (1.6) [47.2, 53.6] | 56.2 (1.5) [53.3, 59.1] | 51.5 (1.5) [48.5, 56.3] |
| Self-Monitoring | 52.3 (1.8) [48.6, 56.0] | 52.3 (2.0) [48.2, 56.3] | 54.2 (1.7) [50.8, 57.6] | 49.8 (1.9) [46.1, 53.6] |
| Flexibility | 58.1 (1.6) [54.9, 61.1] | 54.1 (1.6) [50.8, 57.4] | 57.1 (1.5) [54.2, 60.0] | 52.1 (1.5) [49.1, 55.2] |
| Emotional Control | 57.8 (1.7) [54.4, 61.3] | 55.3 (1.5) [51.8, 58.8] | 58.8 (1.6) [55.2, 61.7] | 52.8 (1.6) [49.6, 56.0] |
| Initiating | 58.7 (1.6) [55.2, 62.1] | 54.7 (1.8) [51.1, 58.4] | 55.6 (1.6) [52.4, 58.7] | 54.2 (1.7) [50.8, 57.6] |
| Working Memory | 57.2 (1.7) [53.8, 60.5] | 54.5 (1.7) [53.8, 51.2] | 55.9 (1.5) [52.8, 59.0] | 54.6 (1.5) [51.6, 57.7] |
| Planning/Organizing | 55.6 (1.9) [51.9, 59.3] | 51.9 (2.1) [47.7, 56.1] | 53.1 (1.7) [49.6, 56.5] | 50.0 (2.0) [46.0, 53.9] |
| Task Monitoring | 50.8 (1.6) [47.6, 54.0] | 50.3 (1.8) [46.7, 53.8] | 52.5 (1.5) [49.5, 55.4] | 50.4 (1.7) [47.1, 53.7] |
| Organization of Materials | 51.9 (1.4) [49.0, 54.8] | 50.0 (1.5) [47.0, 53.0] | 51.8 (1.3) [49.1, 54.5] | 49.9 (1.4) [47.1, 52.7] |

**Table S15** 2×2×7 Follow-up Estimated Marginal Means (self-report; child age: 11-17)

|  | **PRT**  Pre-treatment  M(SE) [95 % CI] | **PRT**  Post-treatment  M(SE) [95 % CI] | **CBT**  Pre-treatment  M(SE) [95 % CI] | **CBT**  Post-treatment  M(SE) [95 % CI] |
| --- | --- | --- | --- | --- |
| BRIEF-II Sub Domain |  |  |  |  |
| Inhibition | 49.3 (2.6) [44.1, 54.5] | 44.0 (2.5) [39.0, 49.0] | 49.2 (2.3) [44.6, 53.8] | 45.6 (2.2) [41.2, 50.0] |
| Self-Monitoring | 47.3 (2.5) [42.2, 52.4] | 43.0 (2.0) [39.0, 46.9] | 47.5 (2.2) [43.0, 52.0] | 43.2 (1.7) [39.7, 46.7] |
| Flexibility | 51.6 (2.9) [45.7, 57.4] | 48.1 (2.5) [43.1, 53.1] | 51.3 (2.8) [46.1, 56.5] | 45.0 (2.2) [40.6, 49.4] |
| Emotional Control | 54.3 (2.4) [49.5, 59.1] | 51.8 (2.5) [46.8, 56.7] | 56.2 (2.1) [52.0, 60.4] | 48.0 (2.2) [43.6, 52.4] |
| Task Completion | 50.7 (2.1) [46.4, 54.9] | 49.2 (2.4) [44.5, 54.0] | 51.8 (1.8) [48.1, 55.5] | 46.1 (2.1) [42.0, 50.4] |
| Working Memory | 46.4 (2.3) [41.8, 51.1] | 45.1 (2.5) [40.1, 50.1] | 48.4 (2.1) [44.3, 52.5] | 44.5 (2.2) [40.1, 48.9] |
| Planning/Organizing | 48.5 (2.1) [44.4, 52.7] | 44.7 (2.3) [40.0, 49.3] | 50.1 (1.8) [46.5, 53.8] | 44.4 (2.0) [40.3, 48.5] |

### Follow-up Estimated Marginal Means (Controls)

**Table S16** 2×2×9 Follow-up Estimated Marginal Means (controls; parent-report; child age: 8-17)

|  | **CTR**  Week 0  M(SE) [95 % CI] | **CTR**  Week 16  M(SE) [95 % CI] |
| --- | --- | --- |
| BRIEF-II Sub Domain |  |  |
| Inhibition | 45.6 (0.8) [44.0, 47.1] | 46.5 (0.9) [44.7, 48.2] |
| Self-Monitoring | 44.7 (0.9) [43.0, 46.4] | 44.6 (0.8) [43.0, 46.3] |
| Flexibility | 45.4 (0.9) [43.6, 47.3] | 45.6 (0.8) [44.0, 47.2] |
| Emotional Control | 46.3 (1.0) [44.4, 48.2] | 45.8 (0.9) [44.0, 47.5] |
| Initiating | 45.8 (0.9) [44.0, 47.6] | 46.5 (0.9) [44.7, 48.3] |
| Working Memory | 46.1 (0.7) [44.7, 47.5] | 46.7 (0.8) [45.0, 48.3] |
| Planning/Organizing | 44.6 (1.1) [42.3, 46.8] | 45.4 (1.0) [43.4, 47.5] |
| Task Monitoring | 47.3 (1.0) [45.2, 49.3] | 47.6 (1.1) [45.4, 49.7] |
| Organization of Materials | 48.5 (1.0) [46.6, 50.5] | 48.3 (0.8) [46.7, 50.0] |

**Table S17** 2×2×7 Follow-up Estimated Marginal Means (controls; self-report; child age: 8-17)

|  | **CTR**  Week 0  M(SE) [95 % CI] | **CTR**  Week 16  M(SE) [95 % CI] |
| --- | --- | --- |
| BRIEF-II Sub Domain |  |  |
| Inhibition | 45.8 (1.2) [43.3, 48.2] | 43.7 (1.2) [41.2, 46.1] |
| Self-Monitoring | 46.3 (1.4) [43.5, 49.1] | 43.9 (1.1) [41.6, 46.2] |
| Flexibility | 44.2 (1.1) [41.9, 46.5] | 42.7 (1.1) [40.5, 44.9] |
| Emotional Control | 45.2 (1.1) [43.1, 47.3] | 44.6 (0.9) [42.7, 46.4] |
| Task Completion | 46.4 (1.1) [44.2, 48.7] | 45.3 (1.2) [43.0, 47.7] |
| Working Memory | 46.4 (1.2) [43.9, 49.0] | 45.1 (1.3) [42.4, 47.8] |
| Planning/Organizing | 46.4 (1.5) [43.4, 49.4] | 45.3 (1.4) [42.5, 48.1] |

## Pre-Treatment Executive Function as a Moderator

### Multiple linear regression tables

These tables include the coefficients, standard errors, t-values, and p-values for all predictors and interactions in the regression models predicting CY-BOCS post-treatment based on CY-BOCS pre-treatment, EF pre-treamtent, and interactions with treatment group.

**Supplementary Table S18** Multiple Linear Regression Results for Parent-Report (child age: 8-17)

| Predictor variable^*^ | *β* | *95 % CI [lower, upper]* | *p* |
| --- | --- | --- | --- |
| Intercept | 17.69 | [16.02, 19.35] | **<.001** |
| CY-BOCS pre-treatment | 0.65 | [0.24, 1.06] | **.002** |
| Treatment Group | -2.65 | [-5.98, 0.68] | .117 |
| BRIEF-II sub-domain |  |  |  |
| Inhibition | 0.02 | [-0.26, 0.29] | .911 |
| Self-Monitoring | -0.02 | [-0.25, 0.21] | .873 |
| Flexibility | 0.01 | [-0.29, 0.31] | .943 |
| Emotional Control | 0.03 | [-0.24, 0.31] | .809 |
| Initiating | 0.27 | [-0.01, .055] | .054 |
| Working Memory | -0.60 | [-0.97, -0.23] | **.002** |
| Planning/Organizing | 0.33 | [-0.01, 0.66] | .054 |
| Task Monitoring | 0.11 | [-0.16, 0.39] | .405 |
| Organization of Materials | -0.13 | [-0.43, 0.18] | .410 |
| Interaction terms |  |  |  |
| Inhibition*Treatment Group | 0.19 | [-0.37, 0.75] | .511 |
| Self-Monitoring*Treatment Group | 0.20 | [-0.28, 0.68] | .411 |
| Flexibility*Treatment Group | 0.39 | [-0.22, 0.99] | .205 |
| Emotional Control*Treatment Group | -0.70 | [-1.25, -0.14] | **.014** |
| Initiating*Treatment Group | 0.18 | [-0.38, 0.74] | .523 |
| Working Memory*Treatment Group | -0.13 | [-0.88, 0.62] | .726 |
| Planning/Organizing*Treatment Group | 0.08 | [-0.58, 0.74] | .807 |
| Task Monitoring*Treatment Group | -0.27 | [-0.82, 0.28] | .329 |
| Organization of Materials*Treatment Group | -0.04 | [-0.64, 0.56] | .895 |

*Note*: *) All predictor variables are centered to have a mean of 0.

**Supplementary Table S19** Multiple Linear Regression Results for Self-Report (child age: 11-17)

| Predictor variable^*^ | *β* | *95 % CI [lower, upper]* | *p* |
| --- | --- | --- | --- |
| Intercept | 17.48 | [15.67, 19.29] | **<.001** |
| CY-BOCS pre-treatment | 0.49 | [0.01-0.97] | **0.044** |
| Treatment Group | -2.88 | [-6.53, 0.76] | .119 |
| BRIEF-II sub-domain |  |  |  |
| Inhibition | -0.13 | [-0.41, 0.15] | .366 |
| Self-Monitoring | -0.17 | [-0.41, 0.07] | .157 |
| Flexibility | 0.01 | [-0.22, 0.25] | .911 |
| Emotional Control | 0.20 | [-0.05, 0.44] | .118 |
| Task Completion | 0.40 | [0.15, 0.66] | **.003** |
| Working Memory | -0.35 | [-0.65, -0.04] | **.025** |
| Planning/Organizing | 0.24 | [-0.08, 0.57] | .142 |
| Interaction terms |  |  |  |
| Inhibition*Treatment Group | 0.42 | [-0.15, 0.99] | .145 |
| Self-Monitoring*Treatment Group | -0.12 | [-0.57, 0.34] | .616 |
| Flexibility*Treatment Group | 0.06 | [-0.36, 0.48] | .791 |
| Emotional Control*Treatment Group | -0.66 | [-1.16, -0.16] | **.011** |
| Task Completion*Treatment Group | 0.53 | [0.01, 1.05] | **.044** |
| Working Memory*Treatment Group | -0.26 | [-0.87, 0.36] | .409 |
| Planning/Organizing*Treatment Group | -0.17 | [-0.82, 0.48] | .605 |

*Note*: *) All predictor variables are centered to have a mean of 0.

### Plots of predictors and interactions

**Supplementary Figure S12** Plot: Working Memory as a Unique Predictor in the Multiple Linear Regression Model (Parent-Report; child age: 8-17)


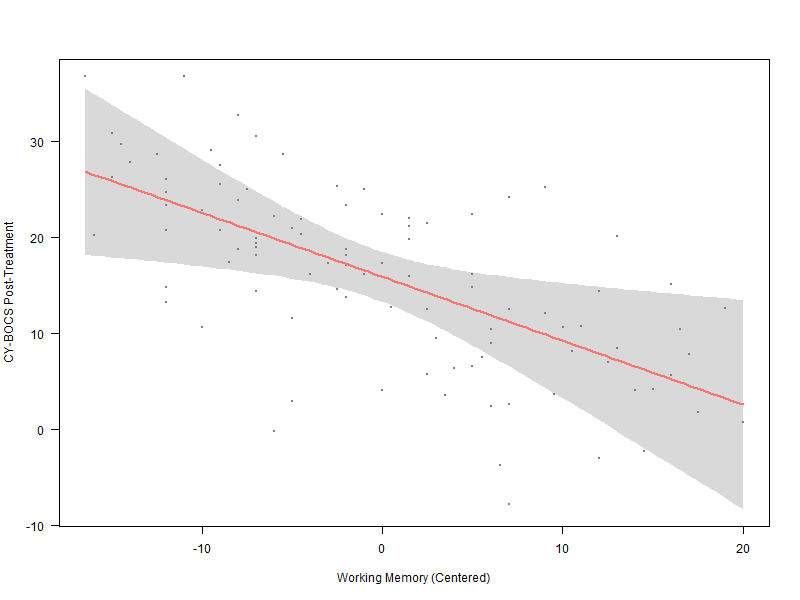


**Supplementary Figure S13** Plot: Emotional Control as a Moderator in the Multiple Linear Regression Model (Parent-Report; child age: 8-17)


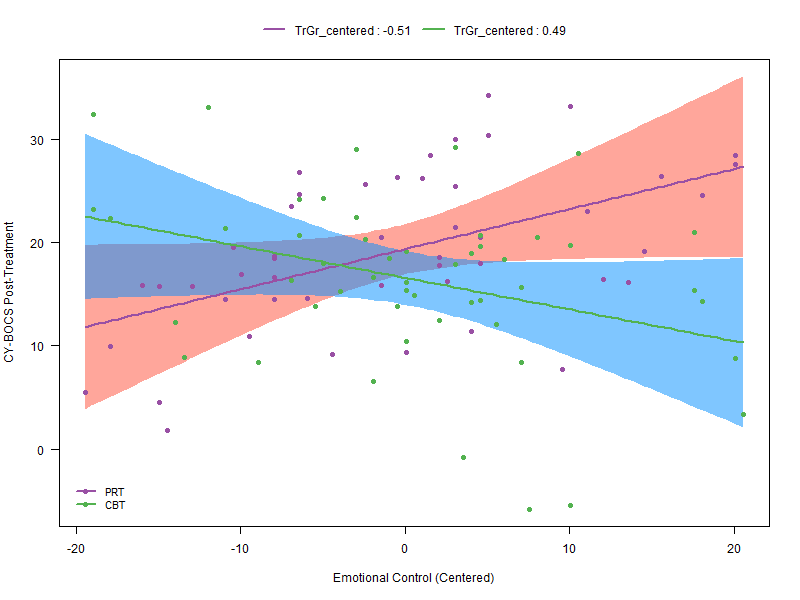


**Supplementary Figure S14** Plot: Working Memory as a Unique Predictor in the Multiple Linear Regression Model (Self-Report; child age: 11-17)


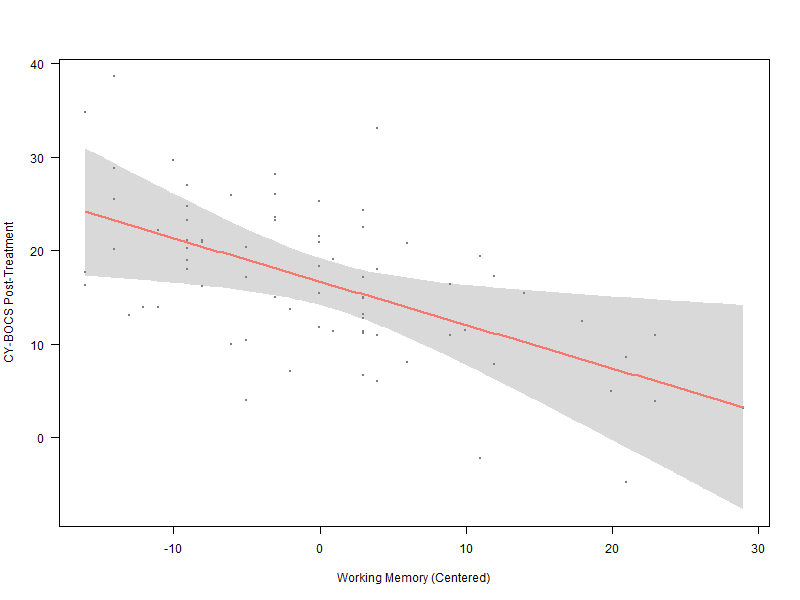


**Supplementary Figure S15** Plot: Task Completion as a Unique Predictor in the Multiple Linear Regression Model (Self-Report; child age: 11-17)


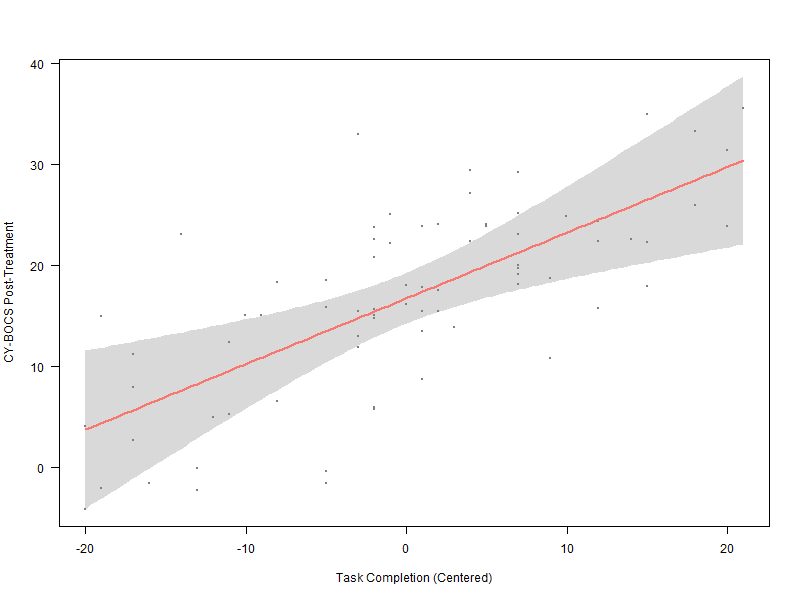


**Supplementary Figure S16** Interaction Plot: Emotional Control as a Moderator (Self-Report; child age: 11-17)


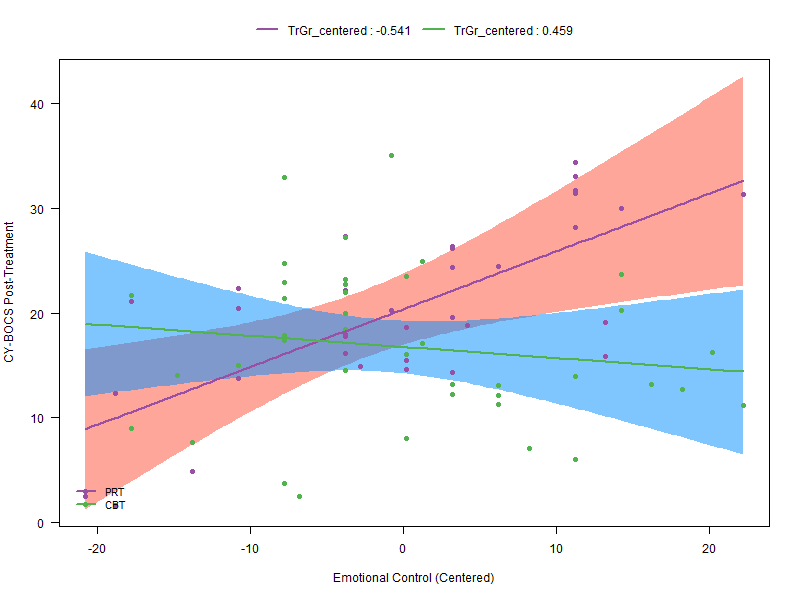


**Supplementary Figure S17** Plot: Task Completion as a Unique Predictor in the Multiple Linear Regression Model (Self-Report; child age: 11-17)


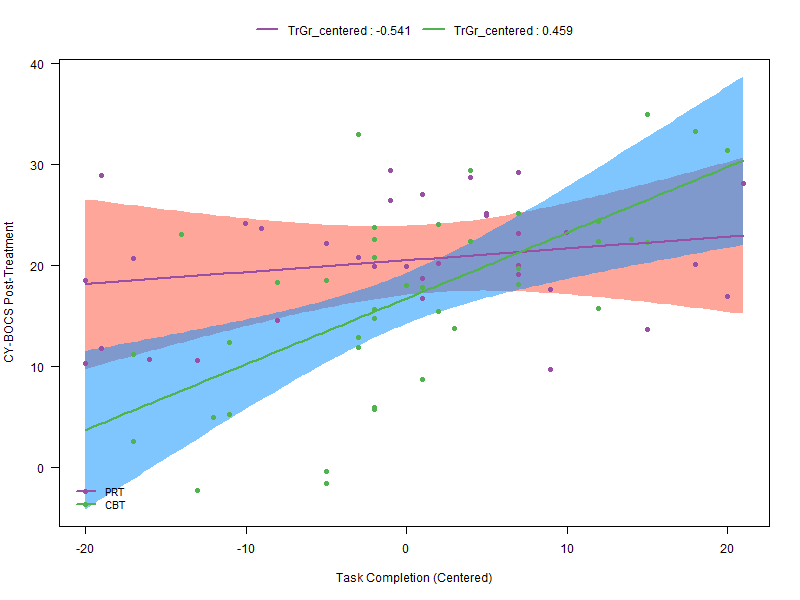

Supplement: Supplementary file 1 — Supplementary Material 1 (DOCX 1.06 MB) [file 787_2026_3013_MOESM1_ESM.docx]
